# Predicting Out-of-Office Blood Pressure in a Diverse US Population

**DOI:** 10.1093/ajh/hpac005

**Published:** 2022-01-18

**Authors:** Brandon K Bellows, Jingyu Xu, James P Sheppard, Joseph E Schwartz, Daichi Shimbo, Paul Muntner, Richard J McManus, Andrew E Moran, Kelsey B Bryant, Laura P Cohen, Adam P Bress, Jordan B King, James M Shikany, Beverly B Green, Yuichiro Yano, Donald Clark, Yiyi Zhang

**Affiliations:** Department of Medicine, Columbia University Irving Medical Center, New York, New York, USA; Department of Medicine, Columbia University Irving Medical Center, New York, New York, USA; Nuffield Department of Primary Care Health Sciences, University of Oxford, Oxford, UK; Department of Medicine, Columbia University Irving Medical Center, New York, New York, USA; Department of Psychiatry and Behavioral Health, Stony Brook University, Stony Brook, New York, USA; Department of Medicine, Columbia University Irving Medical Center, New York, New York, USA; Department of Epidemiology, University of Alabama at Birmingham, Birmingham, Alabama, USA; Nuffield Department of Primary Care Health Sciences, University of Oxford, Oxford, UK; Department of Medicine, Columbia University Irving Medical Center, New York, New York, USA; Department of Medicine, Columbia University Irving Medical Center, New York, New York, USA; Department of Medicine, Columbia University Irving Medical Center, New York, New York, USA; Department of Population Health Sciences, University of Utah, Salt Lake City, Utah, USA; Department of Population Health Sciences, University of Utah, Salt Lake City, Utah, USA; Department of Epidemiology, University of Alabama at Birmingham, Birmingham, Alabama, USA; Kaiser Permanente Washington Health Research Institute, Kaiser Permanente, Seattle, Washington, USA; Department of Family Medicine and Community Health, Duke University, Durham, North Carolina, USA; Department of Medicine, University of Mississippi Medical Center, Jackson, Mississippi, USA; Department of Medicine, Columbia University Irving Medical Center, New York, New York, USA

**Keywords:** ambulatory blood pressure monitoring, blood pressure, blood pressure measurement, cardiovascular disease, high blood pressure, hypertension

## Abstract

**BACKGROUND:**

The PRedicting Out-of-OFfice Blood Pressure (PROOF-BP) algorithm accurately predicted out-of-office blood pressure (BP) among adults with suspected high BP in the United Kingdom and Canada. We tested the accuracy of PROOF-BP in a diverse US population and evaluated a newly developed US-specific algorithm (PROOF-BP-US).

**METHODS:**

Adults with ≥2 office BP readings and ≥10 awake BP readings on 24-hour ambulatory BP monitoring from 4 pooled US studies were included. We compared mean awake BP with predicted out-of-office BP using PROOF-BP and PROOF-BP-US. Our primary outcomes were hypertensive out-of-office systolic BP (SBP) ≥130 mm Hg and diastolic BP (DBP) ≥80 mm Hg.

**RESULTS:**

We included 3,058 adults, mean (SD) age was 52.0 (11.9) years, 38% were male, and 54% were Black. The area under the receiver-operator characteristic (AUROC) curve (95% confidence interval) for hypertensive out-of-office SBP was 0.81 (0.79–0.82) and DBP was 0.76 (0.74–0.78) for PROOF-BP. For PROOF-BP-US, the AUROC curve for hypertensive out-of-office SBP was 0.82 (0.81–0.83) and for DBP was 0.81 (0.79–0.83). The optimal predicted out-of-office BP ranges for out-of-office BP measurement referral were 120–134/75–84 mm Hg for PROOF-BP and 125–134/75–84 mm Hg for PROOF-BP-US. The 2017 American College of Cardiology/American Heart Association BP guideline (referral range 130–159/80–99 mm Hg) would refer 93.1% of adults not taking antihypertensive medications with office BP ≥130/80 mm Hg in the National Health and Nutrition Examination Survey for out-of-office BP measurement, compared with 53.1% using PROOF-BP and 46.8% using PROOF-BP-US.

**CONCLUSIONS:**

PROOF-BP and PROOF-BP-US accurately predicted out-of-office hypertension in a diverse sample of US adults.

## Graphical Abstract

**Graphical Abstract:**
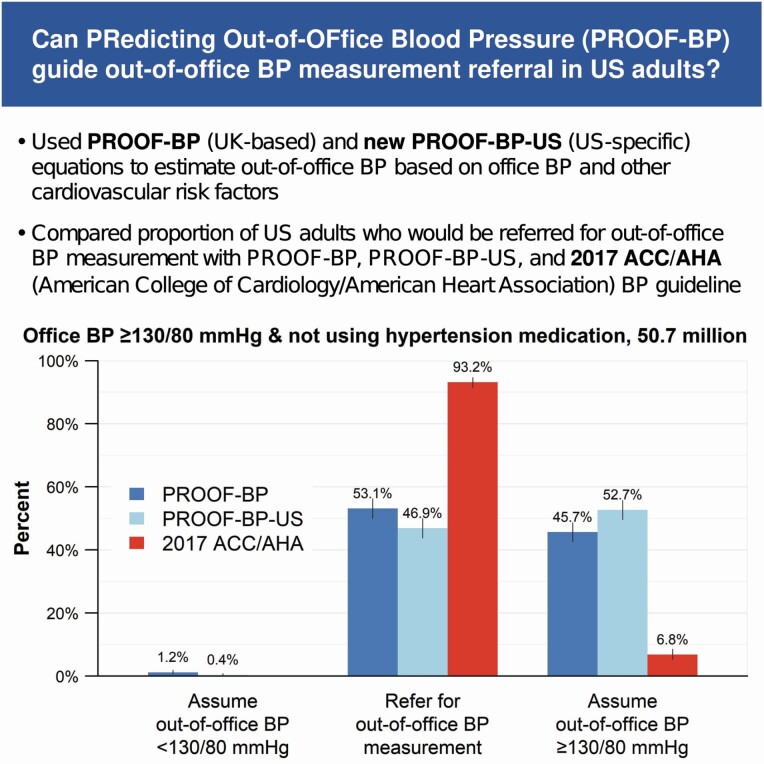

Out-of-office blood pressure (BP) measurements, including home BP monitoring and ambulatory BP monitoring (ABPM), better estimate cardiovascular disease risk than BP measured in the office setting.^1–4^ Out-of-office BP measurements can also be used in conjunction with office BP to identify out-of-office BP phenotypes, such as masked hypertension (i.e., office normotension with out-of-office hypertension) and white coat hypertension (i.e., office hypertension with out-of-office normotension) among individuals not taking antihypertensive medications, which can be used to understand cardiovascular disease risk and guide treatment decisions.^4–16^

The 2017 American College of Cardiology (ACC)/American Heart Association (AHA) BP guideline recommends using out-of-office BP measurement to confirm or exclude a hypertension diagnosis and manage antihypertensive treatment.^4^ Implementation of this recommendation in clinical practice may be costly and logistically challenging, presenting barriers to the adoption of out-of-office BP measurement.^4,17–19^ The PRedicting Out-of-OFfice Blood Pressure algorithm (PROOF-BP), developed and validated in patients from the United Kingdom and Canada, has been shown to accurately predict out-of-office BP based on patient characteristics and office BP readings.^20,21^ PROOF-BP can be used to select patients for out-of-office BP measurement by identifying those with either a high or low likelihood of out-of-office hypertension and thereby reduce the need for potentially time-consuming and burdensome out-of-office BP measurement. However, PROOF-BP was developed primarily in hypertensive individuals (76.0% had a history of hypertension, mean office systolic BP [SBP]/diastolic BP [DBP] 141.3/84.1 mm Hg) and defined out-of-office hypertension using European guidelines (i.e., mean awake SBP/DBP ≥135/85 mm Hg).^22,23^ The 2017 ACC/AHA BP guideline has a lower threshold for defining out-of-office hypertension (i.e., mean awake SBP/DBP ≥130/80 mm Hg), and the ability of PROOF-BP to discriminate between individuals with and without out-of-office hypertension using this threshold is not known.^20^

We assessed the accuracy of PROOF-BP using pooled data from 4 US studies with high-quality office and out-of-office BP measurements. We also derived and internally validated a new algorithm in US adults (PROOF-BP-US) to determine if the accuracy PROOF-BP could be improved. Finally, we used data from the National Health and Nutrition Examination Survey (NHANES) to estimate the number of US adults that would be recommended for out-of-office BP measurement using PROOF-BP, PROOF-BP-US, and the 2017 ACC/AHA BP guideline.

## METHODS

### Population

#### Pooled US studies.

We pooled data from US adults who completed 24-hour ABPM in the (i) Coronary Artery Risk Development in Young Adults (CARDIA) study, (ii) Jackson Heart Study (JHS), (iii) Masked Hypertension Study (MHTS), and (iv) Improving the Diagnosis of Hypertension (IDH) study.^3,24–29^ A description of each study and data collection are included in Supplementary Material online. The data used in our analysis are available upon reasonable request and documentation of human subject protection approval from the Publication Committees for the individual studies. The Institutional Review Boards approved all study protocols at participating institutions, and all participants provided written informed consent.

**Figure 1.**
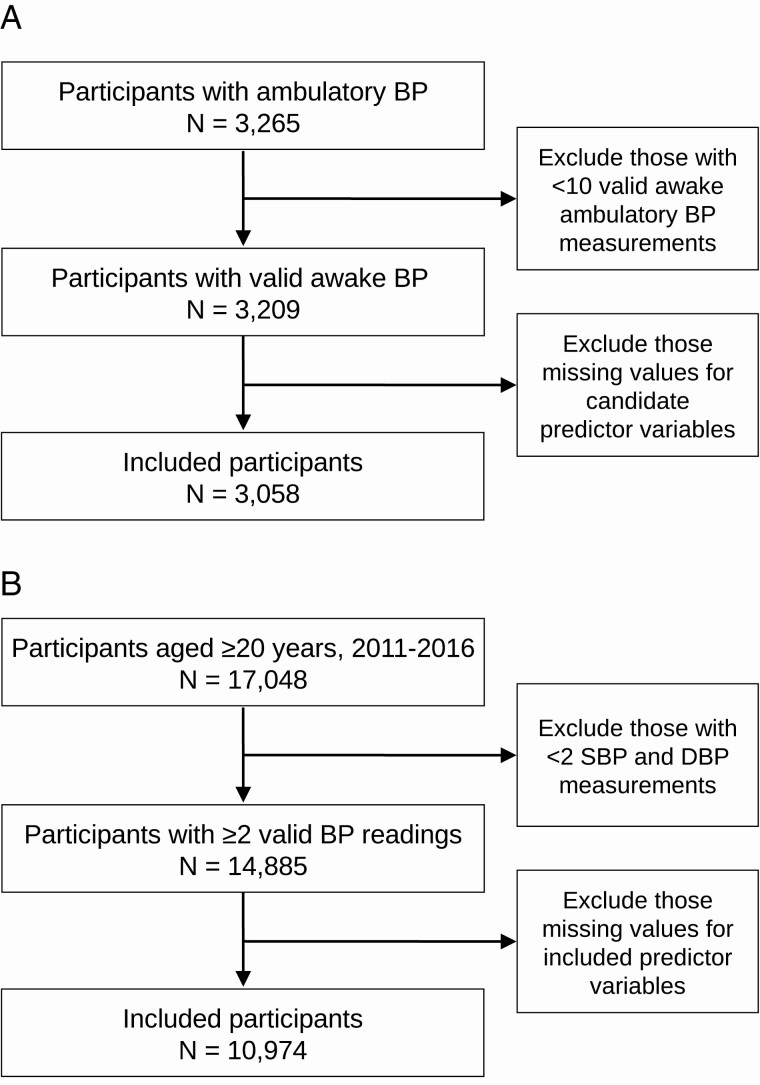
Study population flowcharts. (**a**) Pooled US studies. (**b**) NHANES. Abbreviations: BP, blood pressure; DBP, diastolic blood pressure; NHANES, National Health and Nutrition Examination Survey; PROOF-BP, PRedicting Out-of-OFfice Blood Pressure; SBP, systolic blood pressure. Notes: Panel (a) shows the number of individuals included from the pooled US studies: (i) Coronary Artery Risk Development in Young Adults study, (ii) Jackson Heart Study, (iii) Masked Hypertension Study, and (iv) Improving the Detection of Hypertension study. Panel (b) shows the number of individuals from the 2011 to 2016 NHANES cycles included.

For the current analysis, we included only participants with a complete ABPM recording (Figure 1). All participants had ≥2 office BP readings obtained during a single study visit. We excluded participants with <10 awake BP measurements on ABPM as they did not meet the International Database of Ambulatory Blood Pressure in relation to Cardiovascular Outcomes criteria for a complete awake ambulatory BP recording.^30^ We also excluded participants with missing values for the candidate variables used for PROOF-BP-US (Supplementary Table S1 online). Overall, 3,058 of the 3,265 participants (93.7%) who performed ABPM in the 4 studies met the inclusion criteria for the current analysis.

#### National Health and Nutrition Examination Survey.

NHANES is a cross-sectional national survey that can be weighted to generate prevalence estimates representative of the civilian, noninstitutionalized US population.^31^ We combined the NHANES 2011–2012, 2013–2014, and 2015–2016 cycles to represent the contemporary US population. We included NHANES participants who were ≥20 years old and had ≥2 office BP readings at their study visit. Out-of-office BP measurement was not performed in NHANES. The Institutional Review Board at the National Center for Health Statistics of the Centers for Disease Control and Prevention approved the NHANES protocols, and all participants provided written informed consent. Of the 17,048 participants in NHANES aged ≥20 years, we included 10,974 (64.4%) with complete data needed for the analysis (Figure 1).

### BP classification

Office BP was calculated as the mean of at least 2 office BP readings, with office hypertension defined as SBP or DBP ≥130 or ≥80 mm Hg. Out-of-office SBP and DBP were defined as mean awake ambulatory SBP and DBP, with the awake periods during the 24-hour ABPM defined from actigraphy in CARDIA, IDH, and MHTS, and by self-report when available or a fixed time window (10 am to 8 pm) in JHS. Out-of-office hypertension was defined as mean awake SBP/DBP ≥130/80 mm Hg. Within strata defined by use/nonuse of antihypertensive medications, participants were classified into 1 of 4 out-of-office BP phenotypes: (i) sustained normotension/sustained controlled BP (office and out-of-office normotension), (ii) masked hypertension/masked uncontrolled hypertension (office normotension, out-of-office hypertension), (iii) white coat hypertension/white coat effect (office hypertension, out-of-office normotension), and (iv) sustained hypertension/sustained uncontrolled BP (office and out-of-office hypertension).^4^

### Statistical analysis

#### Original PROOF-BP algorithm.

We applied the PROOF-BP algorithm to the pooled US studies to predict out-of-office SBP and DBP.^20,21^ PROOF-BP uses linear regression functions to predict the difference between the first office BP reading and out-of-office BP. The predicted out-of-office BP is calculated by adding the predicted difference between the first office BP reading and out-of-office BP to the first office BP reading.

#### US-specific PROOF-BP algorithm.

We developed PROOF-BP-US using *k*-fold cross-validation with 10 folds in the pooled US studies. As with PROOF-BP, we developed separate models to predict mean out-of-office SBP and DBP. Multivariable linear regression was used to predict the difference between the first office BP reading and mean out-of-office BP, and this difference was added to the first office BP reading. Candidate predictor variables were considered if they were (i) included in the original PROOF-BP algorithm or identified as plausible predictors of the difference between office and out-of-office BP in a published systematic review of out-of-office BP monitoring studies, and (ii) available in the pooled cohort and NHANES (Supplementary Table S1 online).^20,22^ Age, sex, and first office BP reading were prespecified for inclusion in all models. Other covariates, including squared and interaction terms, were selected for PROOF-BP-US using a multistep backward elimination process, which was performed separately for SBP and DBP (Supplementary Material online). Covariate selection was performed using *k*-fold cross-validation by (i) dividing the pooled US studies into 10 folds, (ii) holding out one of the folds, (iii) selecting covariates based on the 9 included folds, (iv) testing the model performance on the held-out fold, and (v) repeating the process until each of the 10 folds was used as the hold out. From the resulting 10 models, the final covariates were those selected for inclusion in more than half of the 10 models. The final coefficients were calculated by fitting the model to the entire pooled dataset. In sensitivity analysis, the SBP and DBP models were developed including race (Black vs. non-Black) as a candidate variable for inclusion.

#### Model validation.

We externally validated PROOF-BP using the entire pooled dataset and examined the predicted and observed out-of-office BP mean difference, mean absolute difference, Pearson correlation coefficients, and residual plots. We assessed the ability of PROOF-BP to discriminate out-of-office SBP ≥130 mm Hg and out-of-office DBP ≥80 mm Hg using the area under the receiver-operator characteristic (AUROC) curve, and estimated the sensitivity and specificity across a range of predicted out-of-office BPs.^4,20^ We internally validated PROOF-BP-US using a second *k*-fold cross-validation with 10 folds, refitting the final model on the included 9 folds, which produces new coefficients, and predicting the out-of-office SBP and DBP on the held-out fold. The internal validation of PROOF-BP-US was performed on the each of the held-out folds using the same measures as for PROOF-BP.

#### Optimal ranges for out-of-office BP measurement referral.

We sought to determine predicted out-of-office BP ranges from PROOF-BP and PROOF-BP-US that could be used by clinicians to guide out-of-office BP measurement referral. Based on participants’ predicted out-of-office SBP and DBP, the PROOF-BP and PROOF-BP-US recommendations were to: assume out-of-office normotension/controlled BP (i.e., both predicted out-of-office SBP and DBP below the ranges), refer for out-of-office BP measurement (i.e., either predicted out-of-office SBP or DBP within the range), and assume out-of-office hypertension/uncontrolled BP (i.e., either predicted out-of-office SBP or DBP above the range). We tested ranges for predicted out-of-office SBP from 120 to 150 mm Hg and DBP from 70 to 100 mm Hg in 5 mm Hg increments (e.g., predicted out-of-office SBP 120–149, 125–149, and 125–144 mm Hg). The optimal ranges were defined as those that minimized the proportion of participants who would be recommended for out-of-office BP measurement and misclassified <20% of participants not recommended for out-of-office BP measurement (Supplementary Material online). We also examined the proportion of participants who would be recommended for out-of-office BP measurement with PROOF-BP and PROOF-BP-US by out-of-office BP phenotype.

#### Projections to US adult population.

We projected the proportion and number of US adults that would be recommended for out-of-office BP measurement using the PROOF-BP and PROOF-BP-US optimal ranges stratified by office hypertension status and antihypertensive medication use. Also, we compared the proportion and number of US adults recommended for out-of-office BP measurement using the PROOF-BP and PROOF-BP-US to the corresponding proportion and number for whom this would be recommended based on the 2017 ACC/AHA BP guideline (Supplementary Table S2 online).

#### Analysis.

All analyses were performed using R version 4.0.2 (Vienna, Austria). Population characteristics are presented as percentages for categorical measures and means and SDs for continuous measures. We used 500 bootstrapped samples to generate accelerated bias-corrected 95% confidence intervals (95% CIs) for estimates in the pooled US studies, including the β-coefficients for PROOF-BP-US (R packages “boot,” “rsample,” and “coxed”). All NHANES estimates accounted for its complex multistage sampling design and were weighted to represent the US adult population.

## RESULTS

### Participant characteristics

In the pooled US studies (i.e., CARDIA, JHS, MHTS, and IDH), the mean (SD) age of participants was 52.0 (11.9) years, 38.0% were men, and 54.0% were Black (Table 1). Overall, 35.0% of participants self-reported having hypertension, 40.6% had office hypertension (SBP/DBP ≥130/80 mm Hg), the mean office SBP/DBP was 121.7 (16.2)/75.2 (9.6) mm Hg, and mean out-of-office awake SBP/DBP was 127.3 (13.5)/78.5 (8.8) mm Hg.

**Table 1.** Characteristics of pooled US studies overall and by study

Characteristic	Overall (*N* = 3,058)	CARDIA (*N* = 800)	JHS (*N* = 1,049)	IDH (*N* = 389)	MHTS (*N* = 820)
Demographic					
Age (years)	52.0 (11.9)	54.7 (3.7)	59.1 (11.1)	41.2 (13.1)	45.4 (10.3)
Male	1,161 (38.0%)	328 (41.0%)	336 (32.0%)	158 (40.6%)	339 (41.3)
Race/ethnicity					
White	940 (30.7%)	306 (38.2%)	0 (0.0%)	56 (14.4%)	578 (70.5%)
Black	1,651 (54.0%)	494 (61.8%)	1,049 (100.0%)	57 (14.7%)	51 (6.2%)
Hispanic	343 (11.2%)	0 (0.0%)	0 (0.0%)	248 (63.8%)	95 (11.6%)
Other	124 (4.1%)	0 (0.0%)	0 (0.0%)	28 (7.2%)	96 (11.7%)
High school degree	2,742 (89.7%)	759 (94.9%)	829 (79.0%)	340 (87.4%)	814 (99.3%)
Clinical					
Smoking status					
Never	2,018 (66.0%)	449 (56.1%)	698 (66.5%)	318 (81.7%)	553 (67.4%)
Former	714 (23.3%)	230 (28.7%)	240 (22.9%)	38 (9.8%)	206 (25.1%)
Current	326 (10.7%)	121 (15.1%)	111 (10.6%)	33 (8.5%)	61 (7.4%)
Alcohol consumption (any in last year)	2,041 (66.7%)	572 (71.5%)	465 (44.3%)	304 (78.1%)	700 (85.4%)
Body Mass Index (kg/m^2^)	29.8 (6.5)	31.3 (7.1)	31.2 (6.5)	27.5 (5.2)	27.6 (5.3)
Diabetes	479 (15.7%)	153 (19.1%)	260 (24.8%)	17 (4.4%)	49 (6.0%)
Chronic kidney disease	305 (10.0%)	94 (11.8%)	179 (17.1%)	13 (3.3%)	19 (2.3%)
History of cardiovascular disease	170 (5.6%)	60 (7.5%)	110 (10.5%)	0 (0.0%)	0 (0.0%)
BP related					
Self-reported hypertension	1,070 (35.0%)	370 (46.2%)	619 (59.0%)	26 (6.7%)	55 (6.7%)
Taking any antihypertensive medication	949 (31.0%)	326 (40.8%)	623 (59.4%)	0 (0.0%)	0 (0.0%)
Office BP					
SBP	121.7 (16.2)	121.6 (17.4)	127.6 (15.8)	117.2 (15.8)	116.3 (13.0)
DBP	75.2 (9.6)	74.8 (11.0)	74.3 (8.5)	76.7 (10.2)	76.0 (8.9)
Office BP					
<120/<75 mm Hg	1,095 (35.8%)	335 (41.9%)	262 (25.0%)	158 (40.6%)	340 (41.5%)
120–129/75–79 mm Hg	722 (23.6%)	172 (21.5%)	282 (26.9%)	74 (19.0%)	194 (23.7%)
130–139/80–89 mm Hg	797 (26.1%)	177 (22.1%)	300 (28.6%)	107 (27.5%)	213 (26.0%)
≥140/90 mm Hg	444 (14.5%)	116 (14.5%)	205 (19.5%)	50 (12.9%)	73 (8.9%)
Office BP (first reading)					
SBP	121.8 (16.5)	121.3 (17.9)	127.4 (16.2)	117.7 (16.2)	117.1 (13.3)
DBP	75.3 (9.8)	75.3 (11.3)	74.1 (8.8)	76.4 (10.4)	76.3 (9.1)
Office BP change (last* minus first reading)					
SBP	−0.3 (6.7)	0.4 (7.0)	0.4 (6.3)	−0.9 (6.3)	−1.5 (6.6)
DBP	−0.2 (4.7)	−1.0 (4.2)	0.4 (4.9)	0.4 (5.1)	−0.5 (4.5)
Out-of-office BP					
SBP	127.3 (13.5)	130.1 (15.4)	129.5 (13.7)	124.1 (12.0)	123.1 (10.3)
DBP	78.5 (8.8)	81.0 (9.2)	77.6 (9.4)	77.7 (8.0)	77.5 (7.4)

Abbreviations: BP, blood pressure; CARDIA, Coronary Artery Risk Development in Young Adults study; DBP, diastolic blood pressure; IDH, Improving the Detection of Hypertension study; JHS, Jackson Heart Study; MHTS, Masked Hypertension Study; SBP, systolic blood pressure. Notes: Values are mean (SD) or *N* (%). Out-of-office BP is defined as mean awake BP from 24-ambulatory BP monitoring.

*The number of BP readings taken at study visits differed: three were taken for CARDIA, IDH, and MHTS, and two were taken for JHS.

### Validation of PROOF-BP and PROOF-BP-US

The external validation of PROOF-BP found a mean difference between the predicted and observed out-of-office SBP and DBP of −1.4 mm Hg (95% CI −1.8, −1.1) and −1.8 mm Hg (95% CI −2.1, −1.6), respectively, and a mean absolute difference between predicted and observed out-of-office SBP and DBP of 8.1 mm Hg (95% CI 7.9, 8.4) and 6.1 mm Hg (95% CI 5.9, 6.3), respectively (Supplementary Figure S1 online). The Pearson correlation coefficient between predicted and observed out-of-office SBP and DBP with PROOF-BP was 0.62 (95% CI 0.60, 0.65) and 0.52 (95% CI 0.49. 0.55), respectively (Supplementary Figures S2 and S3 online). Residual plots are shown in Supplementary Figures S4 and S5 online. The AUROC for discriminating out-of-office SBP ≥130 mm Hg was 0.81 (95% CI 0.79, 0.82) and out-of-office DBP ≥80 mm Hg was 0.76 (95% CI 0.74, 0.78) for PROOF-BP (Supplementary Figures S6 and S7 online).

Covariates included in the final PROOF-BP-US models for SBP and DBP and their β-coefficients are shown in Supplementary Table S3 online. The internal validation of PROOF-BP-US found a mean difference between the predicted and observed out-of-office SBP and DBP of 0.0 mm Hg (95% CI −0.4, 0.4) and 0.0 mm Hg (95% CI −0.2, 0.3), respectively, and a mean absolute difference between predicted and observed out-of-office SBP and DBP of 7.7 mm Hg (95% CI 7.5, 7.9) and 5.4 mm Hg (95% CI 5.2, 5.5), respectively. For PROOF-BP-US, the correlation between predicted and observed out-of-office SBP was 0.66 (95% CI 0.64, 0.68) and DBP was 0.61 (95% CI 0.59, 0.64). PROOF-BP and PROOF-BP-US predicted out-of-office BPs were highly correlated for both SBP (0.93, *P* < 0.001) and DBP (0.85, *P* < 0.001). For PROOF-BP-US, the AUROC for out-of-office SBP ≥130 mm Hg was 0.82 (95% CI 0.81, 0.83) and for out-of-office DBP ≥80 mm Hg was 0.81 (95% CI 0.79, 0.83).

**Figure 2.**
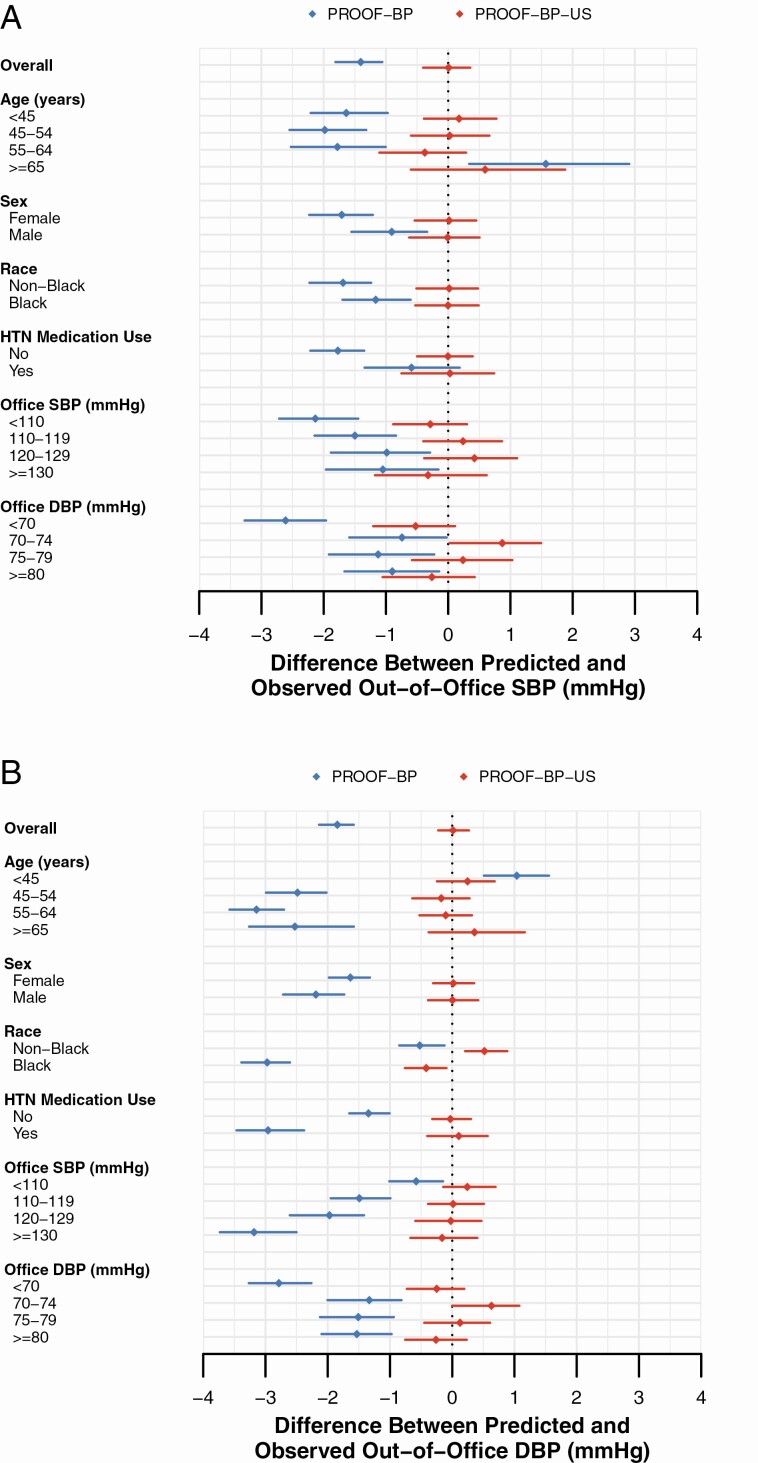
Difference between predicted and observed out-of-office BP overall and in subgroups. (**a**) SBP. (**b**) DBP. Abbreviations: BP, blood pressure; DBP, diastolic blood pressure; PROOF-BP, PRedicting Out-of-OFfice Blood Pressure; SBP, systolic blood pressure. Notes: The figure shows the mean difference (95% confidence interval) between the PROOF-BP and PROOF-BP-US predicted out-of-office blood pressure and the observed out-of-office BP for SBP (panel a) and DBP (panel b).

For PROOF-BP-US, the mean difference between predicted and observed out-of-office SBP and DBP was within ±1 mm Hg for all subgroups examined (Figure 2). Including race as a candidate variable for PROOF-BP-US in sensitivity analysis did not vary the covariates selected for SBP but resulted in different covariates for DBP (Supplementary Table S4 online). However, this did not materially alter identification of out-of-office DBP ≥80 mm Hg (AUROC 0.81).

### Optimal ranges for out-of-office BP measurement referral

Using PROOF-BP, a predicted out-of-office SBP/DBP of 120–134/75–84 mm Hg resulted in the smallest proportion of participants in the pooled US studies who would be referred for out-of-office BP measurement, 61.4% (95% CI 59.7%, 63.0%), and misclassification of 17.4% (95% CI 15.3%, 19.9%) among those who would not be referred for out-of-office BP measurement (Supplementary Figure S8 online). Using PROOF-BP-US, a predicted out-of-office BP of SBP/DBP 125–134/75–84 mm Hg resulted in 58.7% (56.8%, 60.3%) being referred for out-of-office BP measurement, with misclassification of 15.5% (13.7%, 17.6%) among those who would not be referred (Supplementary Figure S9 online). The distribution of participants’ BP phenotype varied by use of antihypertensive medications and predicted out-of-office BP algorithm recommendation (Table 2).

**Table 2.** Observed BP phenotype by PROOF-BP and PROOF-BP-US out-of-office BP measurement recommendations

Out-of-office BP measurement recommendation	Mean percent	Sustained normotension/sustained controlled hypertension	Masked hypertension/masked uncontrolled hypertension	White coat hypertension/white coat effect	Sustained hypertension/sustained uncontrolled hypertension
Not using antihypertensive medications (*N* = 2,109)					
PROOF-BP					
Assume out-of-office normotension	24.3 (22.4–26.1)	84.1 (79.8–86.7)	15.4 (12.4–19.4)	0.4 (0.0–1.0)	0.2 (0.0–0.7)
Refer for out-of-office BP measurement	62.0 (60.2–64.0)	39.1 (36.4–41.5)	23.7 (21.3–25.8)	11.6 (9.8–13.5)	25.6 (23.0–28.0)
Assume out-of-office hypertension	13.7 (12.3–14.9)	2.1 (0.7–3.9)	3.4 (1.4–6.1)	13.2 (9.6–17.7)	81.3 (76.5–85.4)
PROOF-BP-US					
Assume out-of-office normotension	22.5 (20.6–24.3)	85.7 (82.0–88.5)	13.9 (11.2–17.5)	0.0 (0.0–0.0)	0.4 (0.0–1.0)
Refer for out-of-office BP measurement	61.4 (59.4–63.5)	41.4 (38.8–44.1)	24.7 (22.5–27.0)	11.7 (9.9–13.6)	22.1 (19.8–24.2)
Assume out-of-office hypertension	16.1 (14.7–17.6)	1.5 (0.3–3.0)	3.5 (1.5–5.9)	11.8 (8.4–15.0)	83.2 (78.8–86.9)
Using antihypertensive medications (*N* = 949)					
PROOF-BP					
Assume out-of-office controlled BP	7.9 (6.2–9.6)	65.2 (54.5–76.5)	34.8 (23.5–45.5)	0.0 (0.0–0.0)	0.0 (0.0–0.0)
Refer for out-of-office BP measurement	59.9 (57.1–62.5)	36.1 (32.2–40.3)	32 (28.4–35.9)	9.5 (7.4–11.8)	22.4 (19.0–25.9)
Assume out-of-office uncontrolled BP	32.2 (29.5–35.2)	1.0 (0.3–2.6)	1.9 (0.6–3.6)	17.3 (13.0–21.5)	79.8 (75.0–84.0)
PROOF-BP-US					
Assume out-of-office controlled BP	11.1 (9.1–13.1)	74.2 (65.5–81.3)	24.7 (17.0–32.5)	0.0 (0.0–0.0)	1.0 (0.0–5.3)
Refer for out-of-office BP measurement	52.7 (49.8–55.7)	34.8 (31.1–39.0)	35.8 (31.4–40.1)	11.2 (8.5–13.9)	18.2 (15.2–21.8)
Assume out-of-office uncontrolled BP	36.2 (32.8–39.1)	1.4 (0.3–3.0)	2.6 (1.0–4.4)	14.7 (11.0–18.5)	81.3 (76.8–85.5)

Abbreviations: BP, blood pressure; PROOF-BP, PRedicting Out-of-OFfice Blood Pressure. Notes: Values are presented as mean percent (95% confidence interval). BP phenotypes are defined as follows (not using/using antihypertensive medications): sustained normotension/sustained controlled BP—both office and out-of-office BP <130/80 mm Hg, masked hypertension/masked uncontrolled hypertension—office BP <130/80 mm Hg and out-of-office BP ≥130/80 mm Hg, white coat hypertension/white coat effect—office BP ≥130/80 mm Hg and out-of-office BP <130/80 mm Hg, sustained hypertension/sustained uncontrolled BP—both office and out-of-office BP ≥130/80 mm Hg.

### Projections to the US adult population

**Figure 3.**
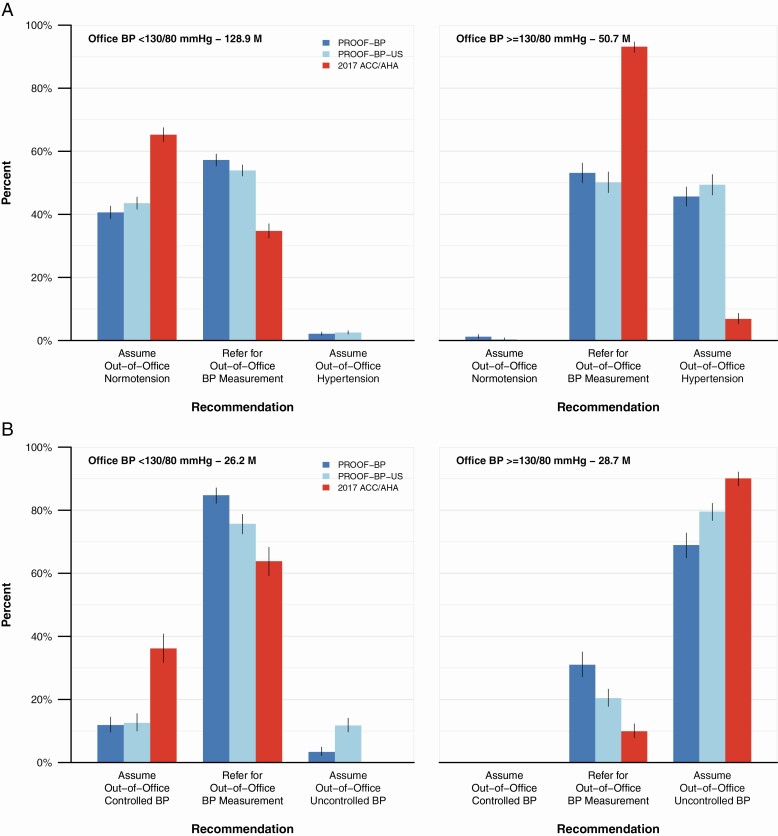
Out-of-office BP measurement recommendations in US adults. (**a**) Not taking antihypertensive medications. (**b**) Taking antihypertensive medications. Abbreviations: ACC, American College of Cardiology; AHA, American Heart Association; BP, blood pressure; PROOF-BP, PRedicting Out-of-OFfice Blood Pressure. Notes: The figure shows the proportion (95% confidence interval) of US adults recommended for out-of-office BP measurement, stratified antihypertensive medication use (panel a—not using antihypertensive medications, panel b—using antihypertensive medications).

Among the 50.7 million US adults not taking antihypertensive medication with an office BP ≥130/80 mm Hg, the 2017 ACC/AHA BP guideline would refer 93.1% (95% CI 91.3%, 94.7%) for out-of-office BP measurement (Figure 3), whereas PROOF-BP would recommend 53.1% (95% CI 50.0%, 56.3%) and PROOF-BP-US 46.8% (95% CI 43.8%, 50.0%). Of the 128.9 million US adults not taking an antihypertensive medication with an office BP <130/80 mm Hg, the proportion referred for out-of-office BP measurement would be 34.7% (95% CI 32.5%, 37.0%) with the 2017 ACC/AHA BP guideline, 57.2% (95% CI 55.3%, 59.2%) with PROOF-BP, and 57.4% (95% CI 55.2%, 59.4%) with PROOF-BP-US. Among individuals using antihypertensive medications, PROOF-BP and PROOF-BP-US would refer a greater proportion for out-of-office BP measurement than the 2017 ACC/AHA guideline.

## DISCUSSION

In this analysis of over 3,000 US adults with both office and out-of-office BP measurements, we externally validated the original PROOF-BP and internally validated the new PROOF-BP-US algorithms. We found that PROOF-BP and PROOF-BP-US discriminated hypertensive out-of-office SBP ≥130 mm Hg equally well, but PROOF-BP-US may offer better discrimination of hypertensive out-of-office DBP ≥80 mm Hg. Compared with the 2017 ACC/AHA BP guideline, using the predicted out-of-office SBP/DBP range of 120–134/75–84 mm Hg for PROOF-BP or 125–134/75–84 mm Hg for PROOF-BP-US would markedly decrease the number of patients referred for out-of-office BP measurement to confirm a diagnosis of hypertension. PROOF-BP and PROOF-BP-US would increase out-of-office BP measurement referrals to identify other BP phenotypes, including masked hypertension.

The new PROOF-BP-US was developed based on PROOF-BP, which was validated in samples from the United Kingdom and Canada.^20,21^ The current analysis differs from the original PROOF-BP analyses in that we (i) used the 2017 ACC/AHA BP guideline thresholds to define out-of-office hypertension, (ii) included a demographically diverse, community-based US population with lower office BPs, and (iii) identified different covariates in the final model. Despite these differences, PROOF-BP and PROOF-BP-US both accurately detected out-of-office hypertension. Another published algorithm showed that masked hypertension could be detected with high sensitivity when office SBP + 1.3*office DBP was between 190 and 217 mm Hg.^32^ While this analysis used a similar population to the current analysis, it was based on the prior guideline thresholds for identifying out-of-office hypertension and was focused only on detecting masked hypertension.

Only about one-quarter of US adults report using home BP monitoring, but a substantial portion of the US adult population would be referred for out-of-office BP measurement based on the 2017 ACC/AHA BP guidelines.^17,32–34^ While BP guidelines and the US Preventive Services Task Force recommend using out-of-office BP measurement to screen for masked and white coat hypertension, there is no consensus on who should be screened.^4,23,35,36^ PROOF-BP and PROOF-BP-US may be used to triage patients referred for out-of-office BP measurement and guide clinical treatment decisions. For example, it could be used to reduce the number of individuals referred for out-of-office BP measurement and allow treatment to begin in those with suspected sustained hypertension while avoiding treatment initiation in those with likely white coat hypertension. Further, PROOF-BP and PROOF-BP-US may also be used to direct out-of-office BP monitoring among those who may have masked hypertension or masked uncontrolled hypertension, which carry a substantial risk for cardiovascular disease.^5–11,15,16^ No randomized clinical trials have been performed to estimate the effect of antihypertensive treatment for masked hypertension, and further research is needed to understand the long-term risks and benefits in this population.

### Strengths and limitations

In this analysis, we leveraged data from 4 US studies to create a large cohort of community-based adults with high-quality office and out-of-office BP measurements. The included participants were demographically and clinically diverse, including a substantial portion without office hypertension who may have masked hypertension or masked uncontrolled hypertension. However, several potential limitations should be considered when interpreting the results. The demographic make-up of the 4 studies included in the analysis was different from the overall US population. These analyses may not be generalizable to larger US populations and smaller subgroups may not be well represented. Our analysis internally validated PROOF-BP-US, and external validation of PROOF-BP-US and direct comparison with the original PROOF-BP in a new sample is needed. We used the 2017 ACC/AHA BP guideline thresholds to define office and out-of-office hypertension, but some organizations in the United States and BP guidelines in other countries use different thresholds.^23,35,37^ However, PROOF-BP performed well in participants with uncontrolled office BP and using higher BP thresholds.^20,21^ Finally, out-of-office BP measurement was performed using ABPM in the pooled US studies, but home BP monitoring is also included in guideline recommendations. The original PROOF-BP showed similar results using either ABPM or home BP monitoring, but the performance of PROOF-BP-US with home BP monitoring has yet to be evaluated.

Using patient characteristics and office BP readings from 4 US studies, both the original PROOF-BP and the US-specific PROOF-BP-US accurately predicted out-of-office SBP and DBP. Both PROOF-BP and PROOF-BP-US may be used to guide clinical decisions and resource allocation among individuals considered for out-of-office BP measurement. Compared with the 2017 ACC/AHA BP guideline, PROOF-BP and PROOF-BP-US could be used to decrease out-of-office BP measurement referrals among patients who may have white coat or sustained hypertension but would increase referrals if also used to identify other BP phenotypes, including masked hypertension.

## Supplementary Material

hpac005_suppl_Supplementary_Material
