# Supplementary material for: Predicting Out-of-Office Blood Pressure in a Diverse US Population
Source: Am J Hypertens. 2022 Jan 18;35(6):533–42. doi: 10.1093/ajh/hpac005 (PMC9203065; doi:10.1093/ajh/hpac005)
Supplement: hpac005_suppl_Supplementary_Material [file hpac005_suppl_supplementary_material.docx]

**SUPPLEMENTAL MATERIAL**

**Title:** Predicting Out-of-Office Blood Pressure in a diverse US population

**Authors:** Brandon K. Bellows, PharmD, MS^1^; Jingyu Xu, MS^1^; James P. Sheppard, PhD^2^; Joseph E. Schwartz, PhD^1,3^; Daichi Shimbo, MD^1^; Paul Muntner, PhD^4^; Richard J. McManus, MA, PhD, MBBS^2^; Andrew E. Moran, MD, MPH^1^; Kelsey B. Bryant, MD, MPH^1^; Laura P. Cohen, MD, MPP^1^; Adam P. Bress, PharmD, MS^5^; Jordan B. King, PharmD, MS^5^; James M. Shikany, DrPH^4^; Beverly B. Green, MD, MPH^6^; Yuichiro Yano, MD^7^; Donald Clark III, MD, MPH^8^; Yiyi Zhang, PhD^1^.

**Affiliations:** (1) Columbia University Irving Medical Center, New York, NY, USA; (2) University of Oxford, Oxford, UK; (3) Stony Brook University, Stony Brook, NY, USA; (4) University of Alabama at Birmingham, Birmingham, AL, USA; (5) University of Utah, Salt Lake City, UT, USA; (6) Kaiser Permanente Washington Health Research Institute, Seattle, WA, USA; (7) Duke University, Durham, NC, USA; (8) University of Mississippi Medical Center, Jackson, MS, USA.

| **Corresponding Author:** |
| --- |
| Brandon K. Bellows, PharmD, MS |
| Department of Medicine, Columbia University |
| 622 West 168th Street, PH9-105 |
| New York, NY 10032 |
| Tel. 212-305-2569 |
| [bkb2132@cumc.columbia.edu](mailto:bkb2132@cumc.columbia.edu) |

**TABLE OF CONTENTS**

**Supplemental Methods – page 3-6**

**Supplemental Figures – pages 7-16**

- Figure S1. Difference and absolute difference between predicted and observed out-of-office BP using PROOF-BP and PROOF-BP-US.
- Figure S2. SBP correlation plots using PROOF-BP and PROOF-BP-US.
- Figure S3. DBP correlation plots using PROOF-BP and PROOF-BP-US.
- Figure S4. Residual SBP plots using PROOF-BP and PROOF-BP-US.
- Figure S5. Residual DBP plots using PROOF-BP and PROOF-BP-US.
- Figure S6. Discrimination of hypertensive out-of-office SBP.
- Figure S7. Discrimination of hypertensive out-of-office DBP.
- Figure S8. Out-of-office BP measurement referral and misclassification rate with PROOF-BP.
- Figure S9. Out-of-office BP measurement referral and misclassification rate with PROOF-BP-US.

**Supplemental Tables – pages 17-22**

- Table S1. Covariates included in PROOF-BP and candidate covariates considered for PROOF-BP-US.
- Table S2. 2017 ACC/AHA out-of-office BP measurement recommendations.
- Table S3. PROOF-BP-US beta coefficients from linear regression models estimating the difference between out-of-office BP and first office BP reading.
- Table S4. Sensitivity analysis: PROOF-BP-US beta coefficients from linear regression models estimating the difference between out-of-office BP and first office BP reading when including race as a potential predictor.

**References – page 23-24**

**SUPPLEMENTAL METHODS**

**Pooled US Studies**

The Coronary Artery Risk Development in Young Adults (CARDIA) is a prospective cohort study that enrolled 5,115 young adults, 18 to 30 years old at baseline, 1985-1986, from four communities across the US (Birmingham, AL; Minneapolis, MN; Chicago, IL; and Oakland, CA) to examine the development of clinical and subclinical cardiovascular disease and their risk factors.^1^ Following the Year 30 Exam (2015-2016), 831 non-pregnant participants underwent ambulatory blood pressure monitoring (ABPM) as part of an ancillary study.^2,3^ The Jackson Heart Study (JHS) is a community-based cohort study of 5,306 African Americans aged 21 years and older, recruited from 2000 to 2004, designed to evaluate the etiology of cardiovascular, renal, and respiratory diseases among African Americans from Jackson, MS.^4^ In JHS, 1148 participants underwent ABPM following the baseline exam. The Masked Hypertension Study (MHTS) enrolled a community sample of 1,011 healthy employed adults between 2005 and 2012 from four worksites in the New York, NY Metropolitan area, of whom 889 completed ABPM, in order to examine the prevalence and correlates of masked hypertension and the difference between office and out-of-office blood pressure (BP).^5^ The Improving the Diagnosis of Hypertension (IDH) Study enrolled a community-based sample of 408 adults, recruited from New York, NY between 2011 and 2013, and was designed to evaluate the reproducibility of alternative methods of assessing BP and strategies for diagnosing hypertension.^6^

**Data Collection**

We used self-report to define demographic information, including age, race/ethnicity, sex, and education in both the pooled cohort and the National Health and Nutrition Examination Survey (NHANES).^1,4,6-8^ Smoking status, alcohol consumption, diagnosis of hypertension, years since hypertension diagnosis, use of antihypertensive medication, and history of cardiovascular disease (i.e., coronary heart disease, heart attack, angina, stroke, and/or heart failure) were also based on self-report.^1,4,6-8^ Body mass index (BMI) was calculated from height and weight, measured according to a standard protocol in each cohort. Diabetes was defined as a fasting glucose ≥126 mg/dL, hemoglobin A1c ≥6.5% (except CARDIA), use of glucose-lowering medication, or by self-report. Chronic kidney disease was defined as estimated glomerular filtration rate <60 mL/min/1.73 m^2^ or albumin-to-creatinine ratio >30 mg/g.

**Change in Office BP**

The original PRedicting Out-of-OFfice BP (PROOF-BP) algorithm included as a predictor the change in office BP from the first to the third BP readings taken at a single office visit. However, JHS had only two BP readings at each study visit, so we used the difference between the second and first office BP reading for JHS participants.

**US-Specific PROOF-BP Algorithm Covariate Selection**

First, we used linear regression to identify variables associated with the difference between the first office BP and ABPM with a p-value <0.10 when adjusting for age, sex, and first office BP reading. Second, we included all variables identified from the first step in a multivariable linear regression model and used backward elimination to identify a parsimonious set of independent predictors, each with a p-value <0.10. Third, we included squared terms for all continuous variables remaining in the model to allow for potential non-linear association with mean out-of-office BP and used backward elimination to keep only those with a p-value <0.10. Fourth, we examined selected interactions (i.e., by age, sex, and office hypertension status) with variables remaining in the model from the second step and used backward elimination to keep those with a p-value <0.10. Finally, to create a parsimonious model, we combined all covariates identified in steps three and four and used backward elimination to include only those with a p-value <0.05.

**Misclassification Rate**

We calculated the misclassification rate as the sum of false positives and false negatives divided by the total number of pooled US studies participants not recommended for out-of-office BP measurement. False positives were those with predicted out-of-office SBP or DBP greater than or equal to the upper thresholds for being referred for out-of-office BP measurement, therefore not referred as assumed to have out-of-office hypertension, but with both an observed out-of-office SBP <130 mmHg and DBP <80 mmHg. False negatives were those with both predicted out-of-office SBP and DBP less than the lower threshold for being referred for out-of-office BP measurement, therefore not referred and assumed to have out-of-office normotension/controlled BP, but with observed out-of-office hypertension.

***Classification of pooled US studies participants based on predicted and observed out-of-office BP.***

| **Predicted out-of-office SBP/DBP** | **Observed out-of-office SBP/DBP** | |
| --- | --- | --- |
|  | *≥130/80 mmHg* | *<130/80 mmHg* |
| *Greater than or equal to upper threshold* | True positive (TP) | False positive (FP) |
| *Within referral range* | Referred | |
| *Less than lower threshold* | False negative (FN) | True Negative (TN) |

$$Misclassification rate=\frac{FP+FN}{TP+FP+FN+TN}$$

We used a <20% misclassification threshold in the population not referred for out-of-office BP measurement to determine the optimal predicted out-of-office BP thresholds. The original PROOF-BP algorithm used a misclassification rate of ≤10% in the total population.^9^ When accounting for the 58% of the population referred for out-of-office BP measurement with PROOF-BP, this corresponds to an error rate of 17.2% in the population not referred. We therefore decided to use <20% among those not referred in the current study.

**SUPPLEMENTAL FIGURES**

**Figure S1. Difference and absolute difference between predicted and observed out-of-office BP using PROOF-BP and PROOF-BP-US.**

| **A) SBP – Difference** | **B) SBP – Absolute Difference** |
| --- | --- |
| 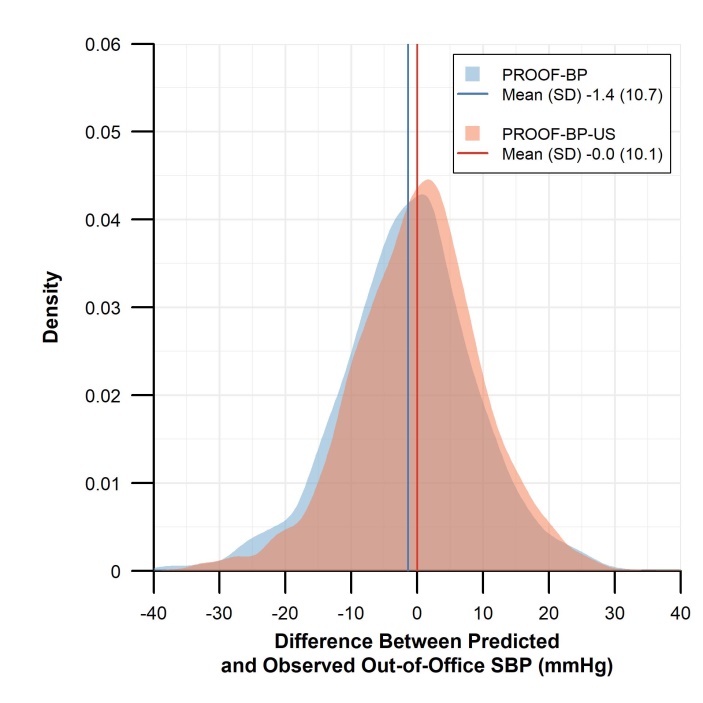 | 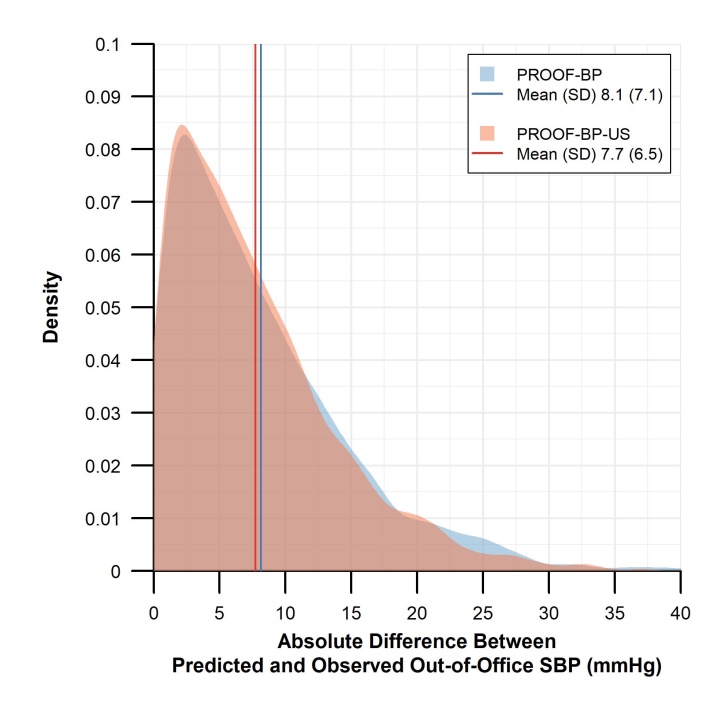 |
| **C) DBP – Difference** | **D) DBP – Absolute Difference** |
| 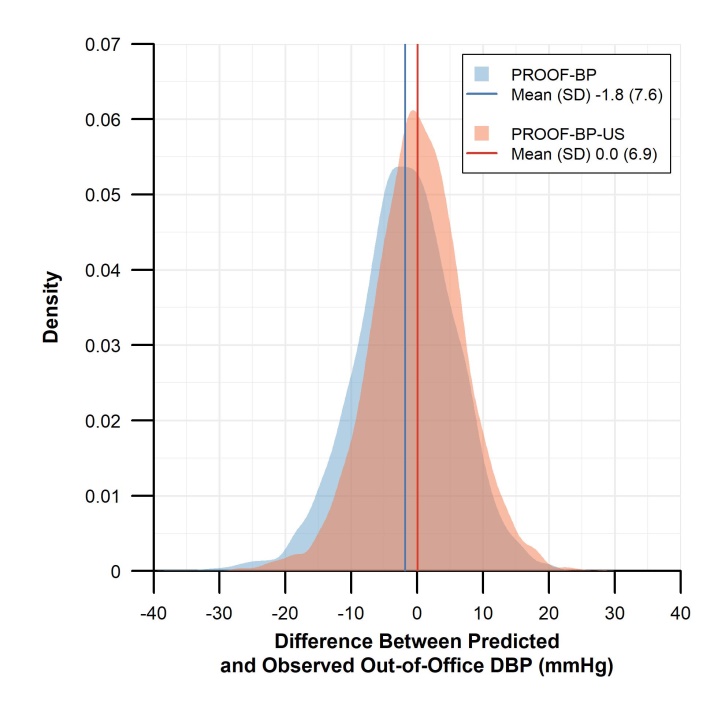 | 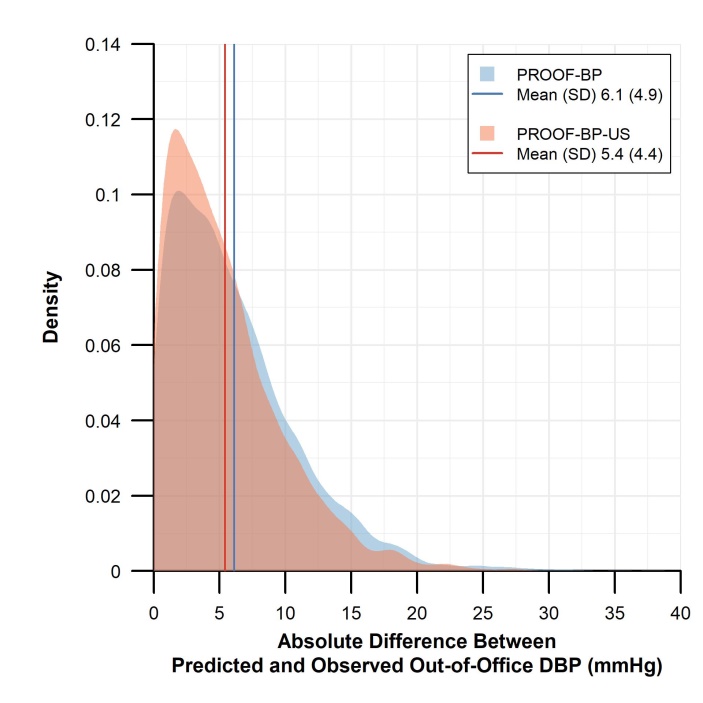 |

DBP – diastolic blood pressure, PROOF-BP – PRedicting Out-of-OFfice Blood Pressure, SBP – systolic blood pressure.

Notes: Mean difference between predicted and observed out-of-office SBP (vertical lines) was -1.4 mmHg (95%CI -1.8 to -1.1 mmHg) for PROOF-BP and -0.0 mmHg (95%CI -0.4 to 0.4 mmHg) with PROOF-BP-US (**Panel A**). Mean absolute difference between predicted and observed out-of-office SBP (vertical lines) was 8.1 mmHg (95%CI 7.9 to 8.4 mmHg) with PROOF-BP and 7.7 mmHg (95%CI 7.5 to 7.9 mmHg) with PROOF-BP-US (**Panel B**). Mean difference between predicted and observed out-of-office DBP (vertical lines) was -1.8 mmHg (95%CI -2.1 to -1.6 mmHg) with PROOF-BP and 0.0 mmHg (95%CI -0.2 to 0.3 mmHg) with PROOF-BP-US (**Panel C**). Mean absolute difference between predicted and observed out-of-office DBP (vertical lines) was 6.1 mmHg (95%CI 5.9 to 6.3 mmHg) with PROOF-BP and 5.4 mmHg (95%CI 5.2 to 5.5 mmHg) with PROOF-BP-US (**Panel D**).

**Figure S2. SBP correlation plots using PROOF-BP and PROOF-BP-US.**

| **A) PROOF-BP** | **B) PROOF-BP-US** |
| --- | --- |
| **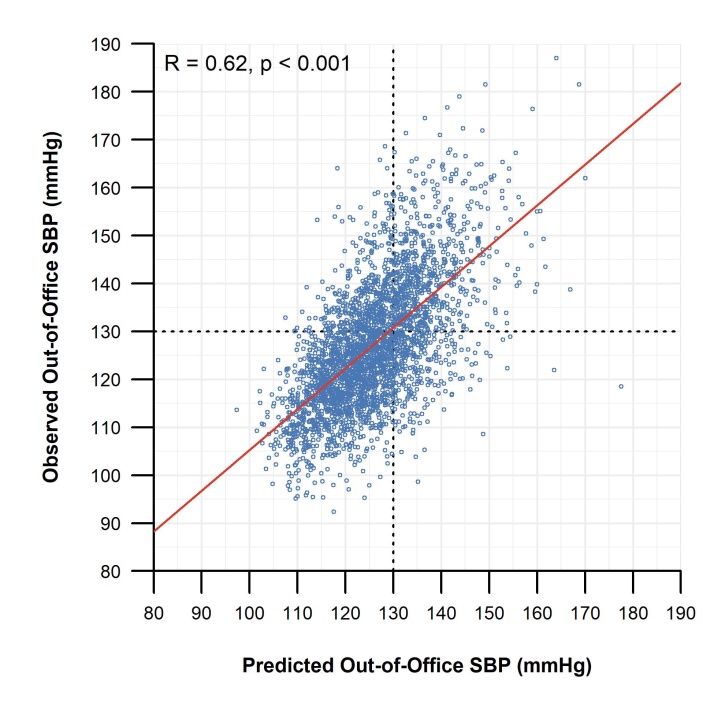** | **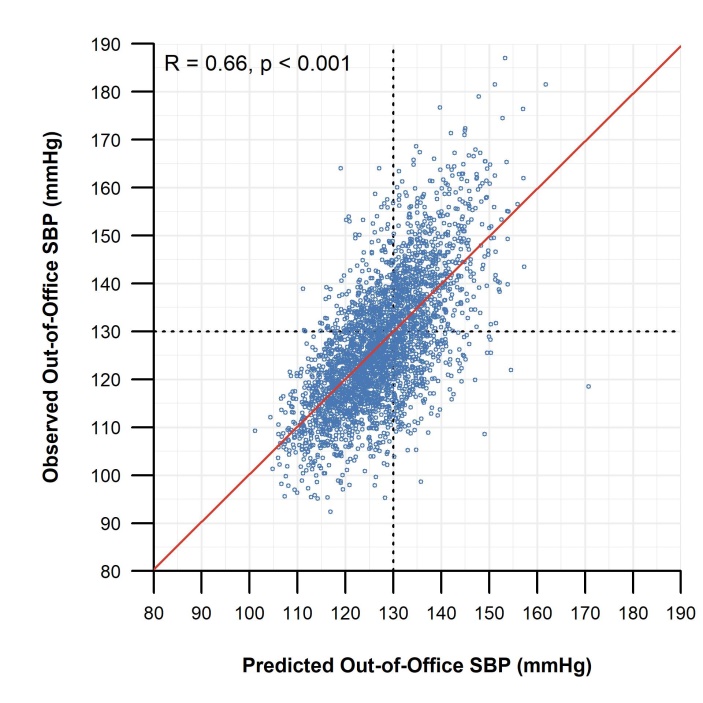** |
| **C) PROOF-BP vs. PROOF-BP-US** |  |
| **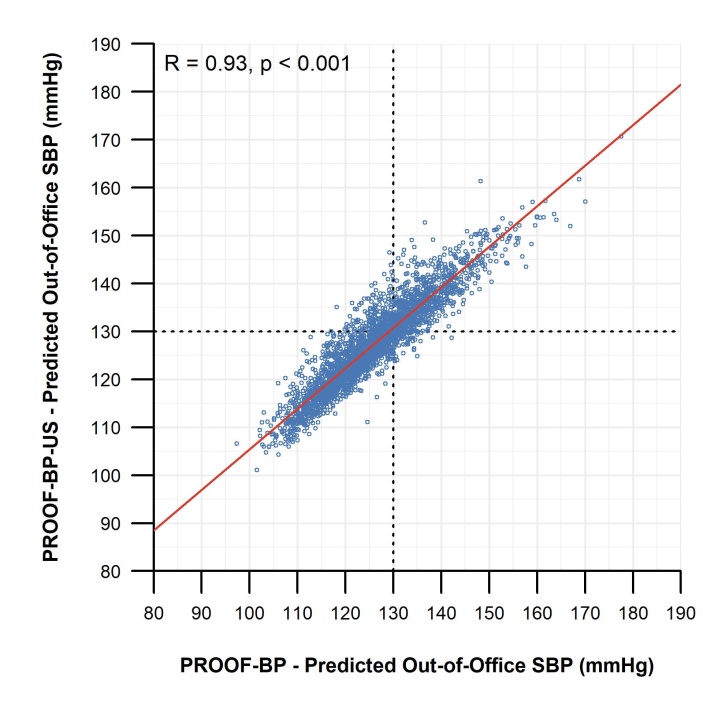** |  |

PROOF-BP – PRedicting Out-of-OFfice Blood Pressure, SBP – systolic blood pressure.

Notes: Pearson correlation coefficient between predicted and observed out-of-office SBP was 0.62 (95%CI 0.60 to 0.65) for PROOF-BP (**Panel A**) and 0.66 (95%CI 0.64 to 0.68) for PROOF-BP-US (**Panel B**). Predicted out-of-office SBPs for PROOF-BP and PROOF-BP-US were highly correlated 0.93 (95%CI 0.92 to 0.93, p<0.001) (**Panel C**).

**Figure S3. DBP correlation plots using PROOF-BP and PROOF-BP-US.**

| **A) PROOF-BP** | **B) PROOF-BP-US** |
| --- | --- |
| **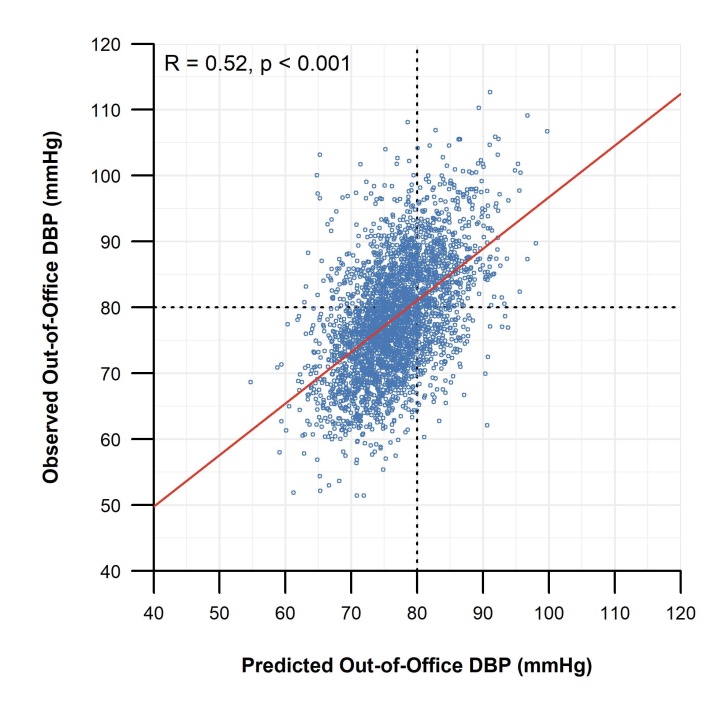** | **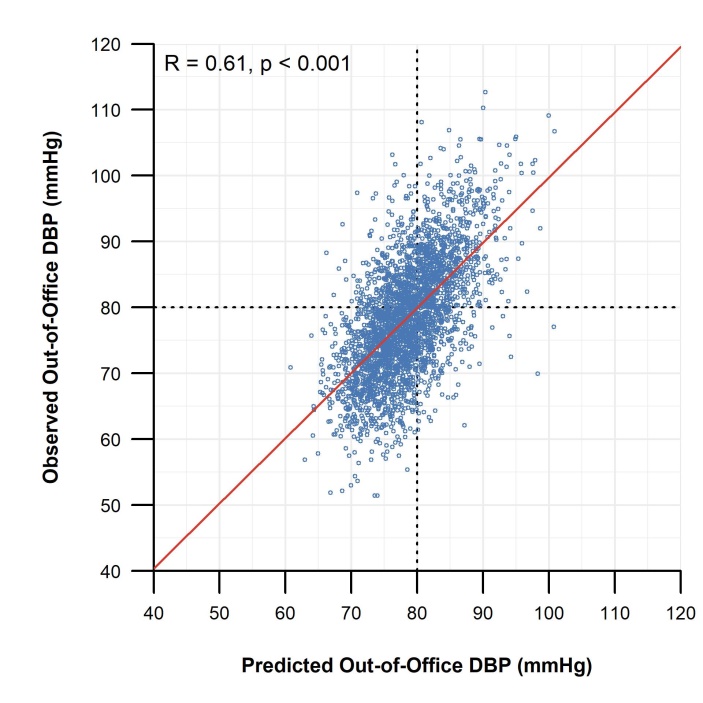** |
| **C) PROOF-BP vs. PROOF-BP-US** |  |
| **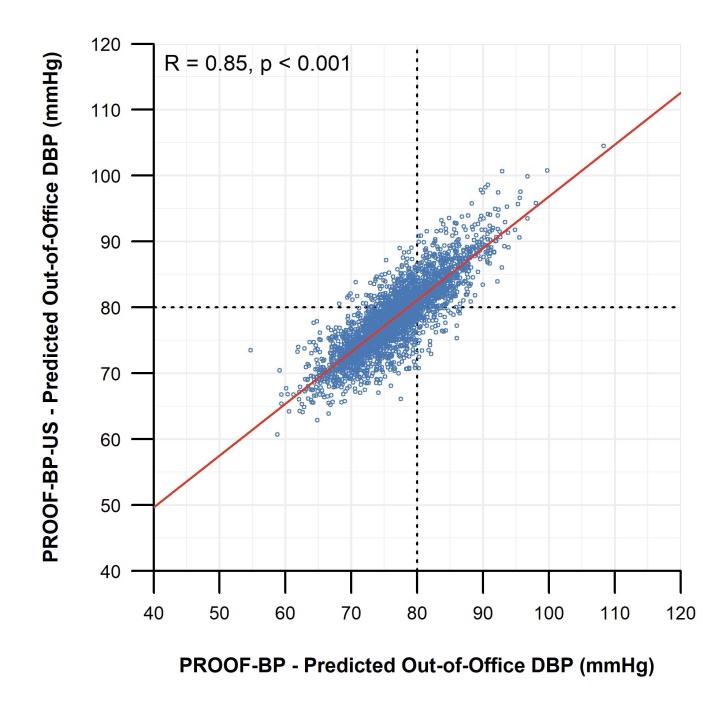** |  |

DBP – diastolic blood pressure, PROOF-BP – PRedicting Out-of-OFfice Blood Pressure.

Notes: Pearson correlation coefficient between predicted and observed out-of-office DBP was 0.52 (95%CI 0.49 to 0.55) for PROOF-BP (**Panel A**) and 0.61 (95%CI 0.59 to 0.64) for PROOF-BP-US (**Panel B**). Predicted out-of-office DBPs for PROOF-BP and PROOF-BP-US were highly correlated 0.85 (95%CI 0.84 to 0.86, p<0.001) (**Panel C**).

.**Figure S4. Residual SBP plots using PROOF-BP and PROOF-BP-US.**

| **A) PROOF-BP** | **B) PROOF-BP-US** |
| --- | --- |
| **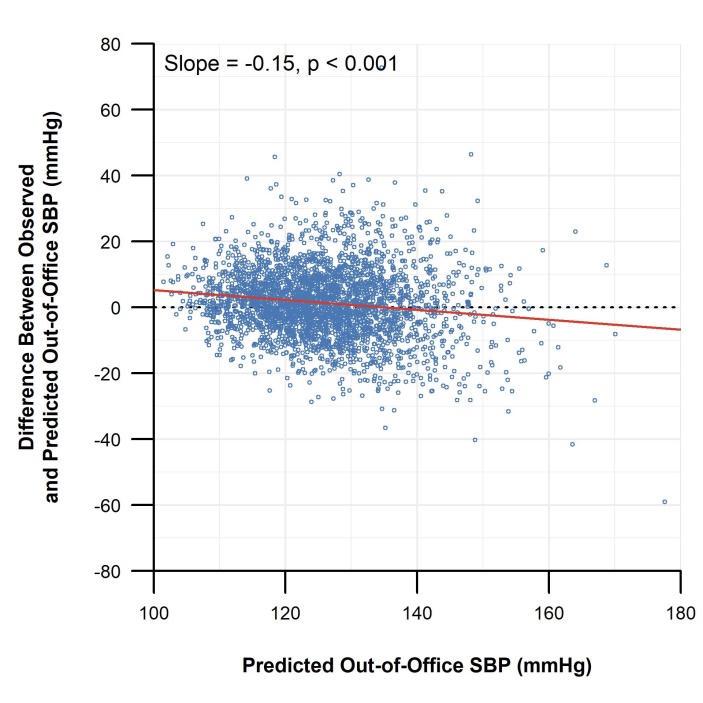** | **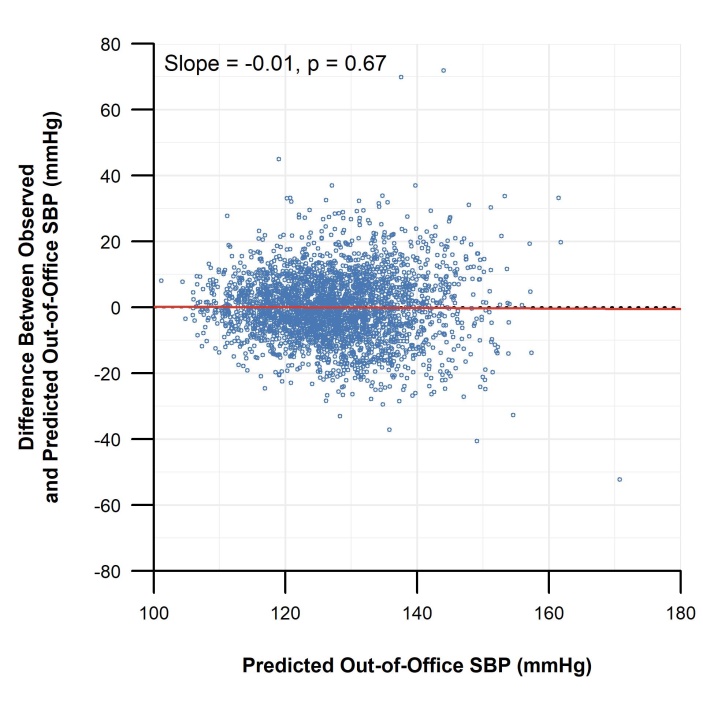** |

PROOF-BP – PRedicting Out-of-OFfice Blood Pressure, SBP – systolic blood pressure.

Notes: The figure shows residual difference between the predicted and observed out-of-office SBP by the predicted out-of-office SBP for PROOF-BP (**Panel A**) and PROOF-BP-US (**Panel B**).

**Figure S5. Residual DBP plots using PROOF-BP and PROOF-BP-US.**

| **A) PROOF-BP** | **B) PROOF-BP-US** |
| --- | --- |
| 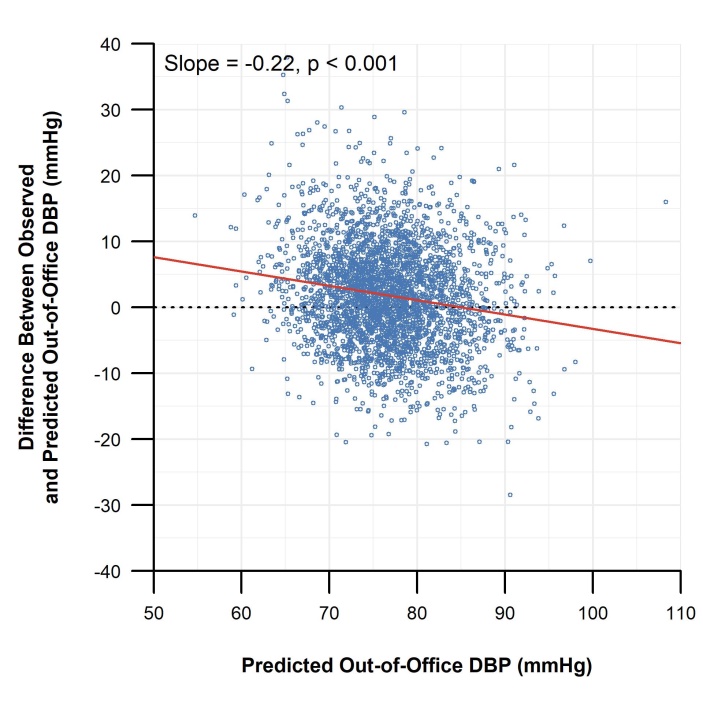 | 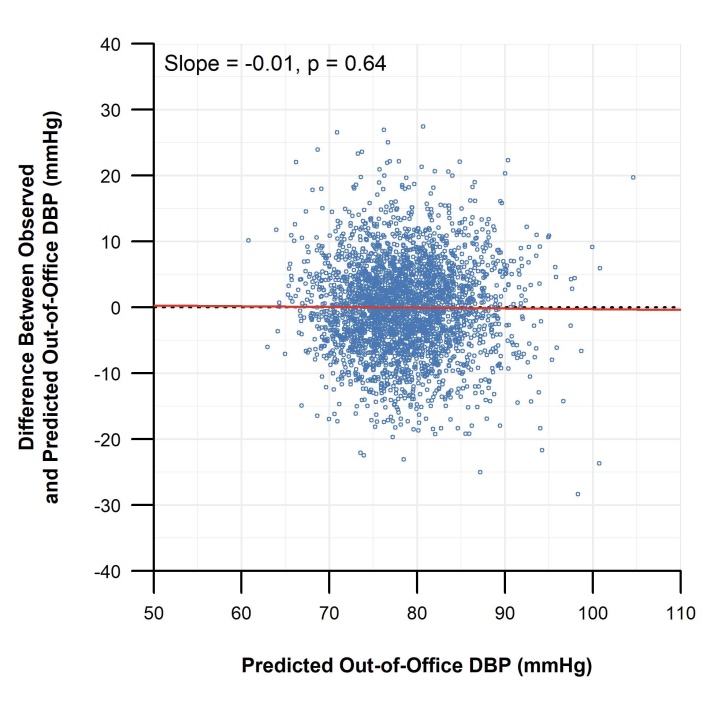 |

DBP – diastolic blood pressure, PROOF-BP – PRedicting Out-of-OFfice Blood Pressure.

Notes: The figure shows residual difference between the predicted and observed out-of-office DBP by the predicted out-of-office DBP for PROOF-BP (**Panel A**) and PROOF-BP-US (**Panel B**).

**Figure S6. Discrimination of hypertensive out-of-office SBP.**

| **A) AUROC** | **B) Sensitivity and Specificity** |
| --- | --- |
| 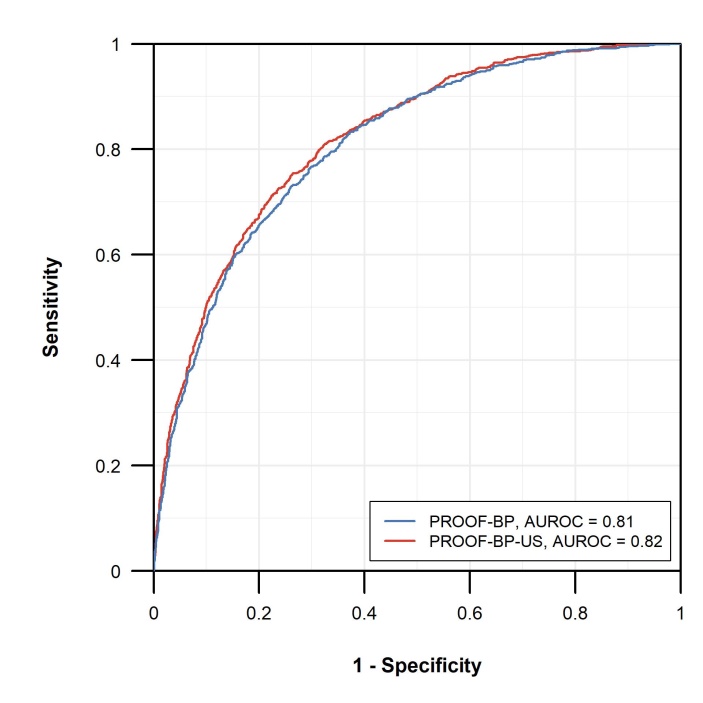 | 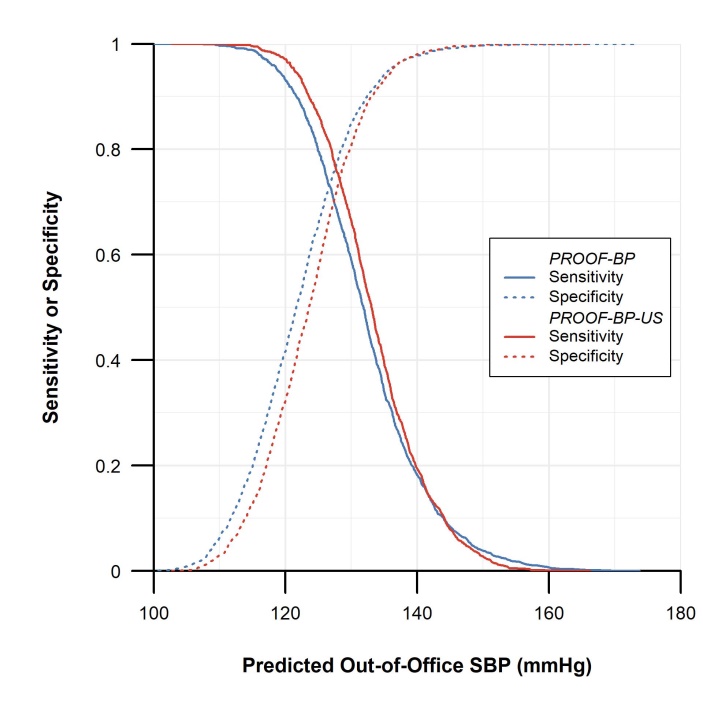 |

AUROC – area under the receiver-operator characteristic, PROOF-BP – PRedicting Out-of-OFfice Blood Pressure, SBP – systolic blood pressure.

Notes: Hypertensive out-of-office SBP is mean awake SBP ≥130 mmHg. The figure shows the AUROC for PROOF-BP and PROOF-BP-US (**Panel A**) and the sensitivity and specificity for different thresholds of predicted out-of-office SBP (**Panel B**).

**Figure S7. Discrimination of hypertensive out-of-office DBP.**

| **A) AUROC** | **B) Sensitivity and Specificity** |
| --- | --- |
| 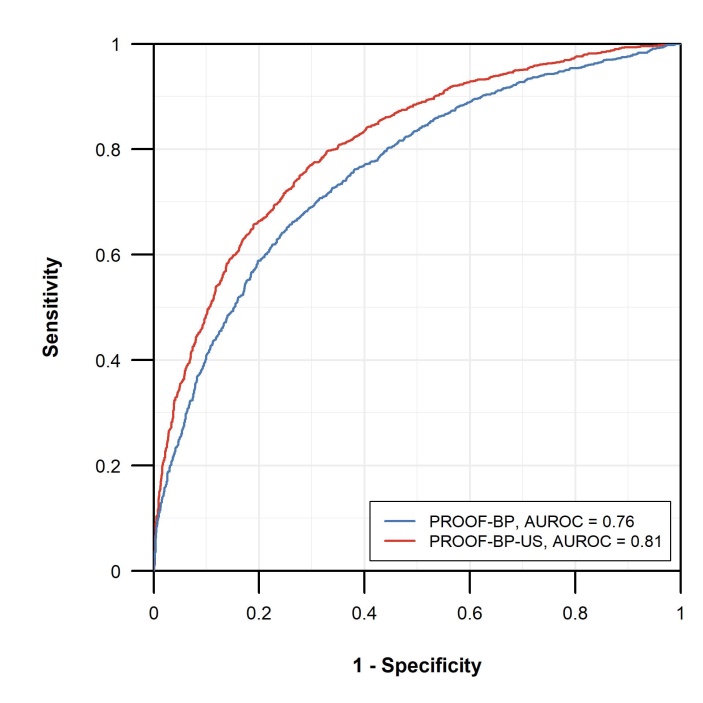 | 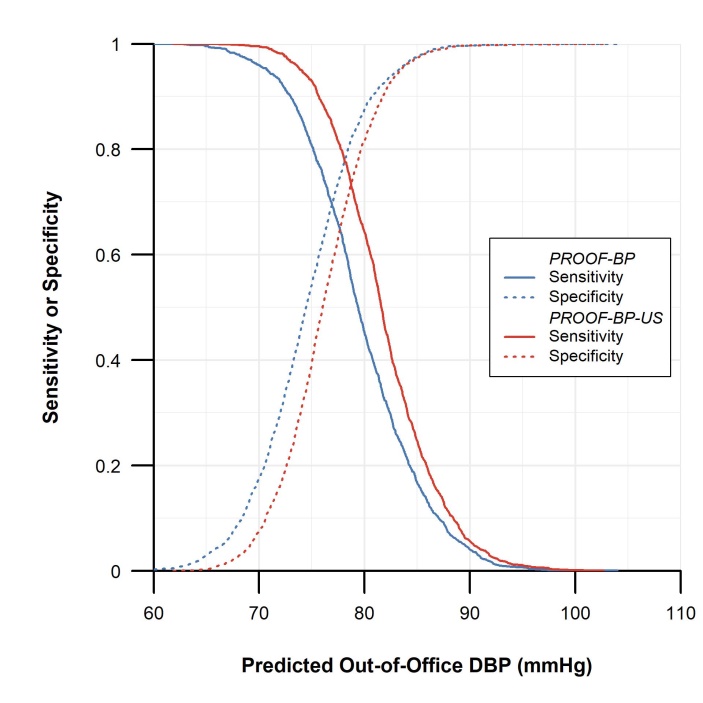 |

AUROC – area under the receiver-operator characteristic, DBP – diastolic blood pressure, PROOF-BP – PRedicting Out-of-OFfice Blood Pressure.

Notes: Hypertensive out-of-office DBP is mean awake DBP ≥80 mmHg. The figure shows the AUROC for PROOF-BP amd PROOF-BP-US (**Panel A**) and the sensitivity and specificity for different thresholds of predicted out-of-office DBP (**Panel B**).

**Figure S8. Out-of-office BP measurement referral and misclassification rate with PROOF-BP.**

| **A) Proportion recommended for out-of-office BP measurement** | **B) Misclassification rate among participants not recommended for out-of-office BP measurement** | |  |
| --- | --- | --- | --- |
| **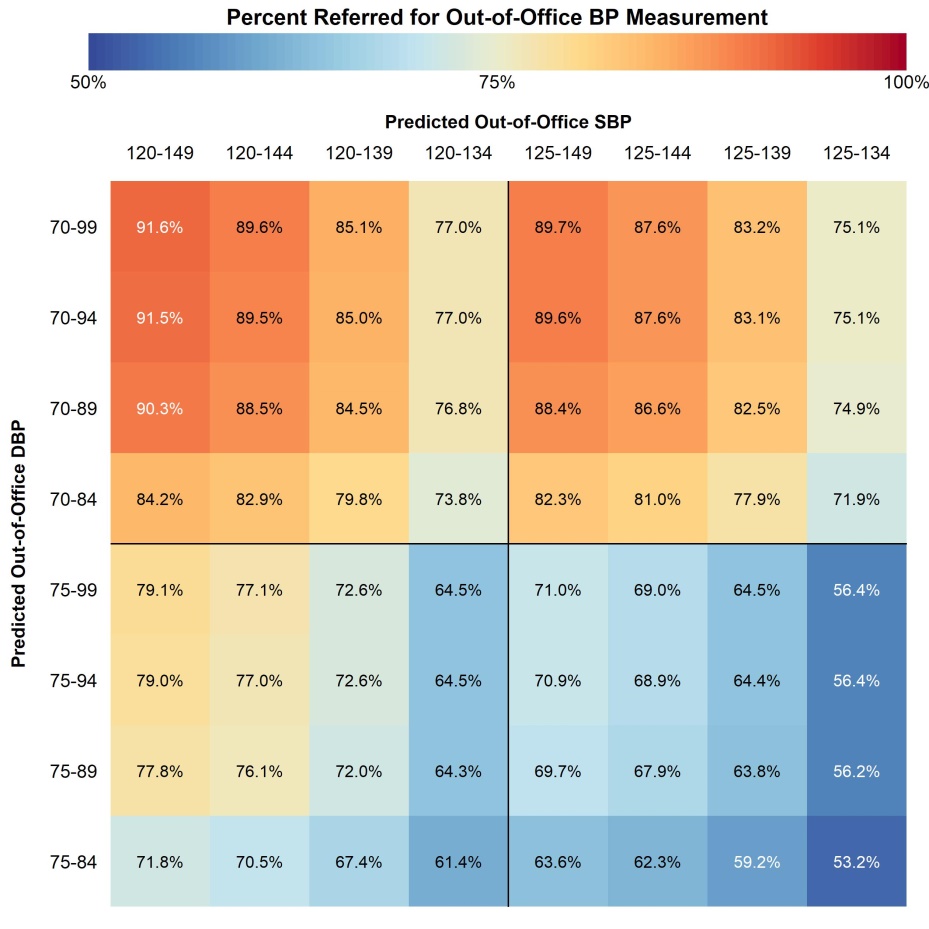** | | **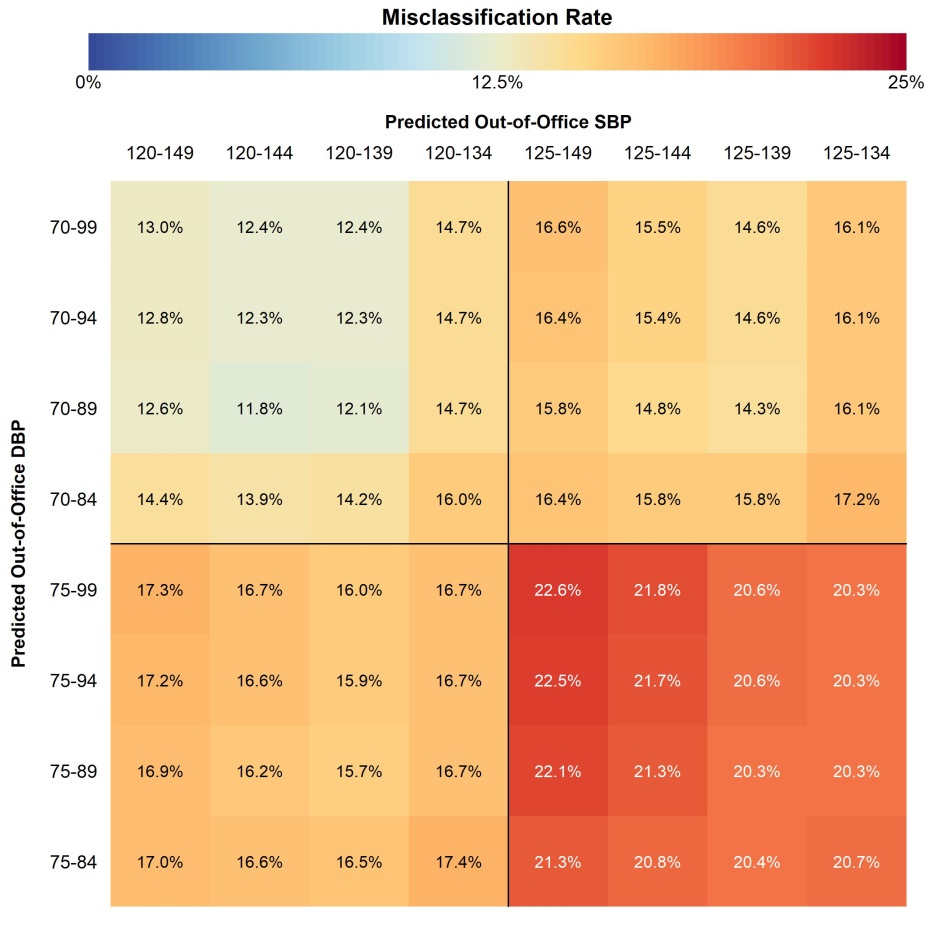** | |

BP – blood pressure, DBP – diastolic blood pressure, PROOF-BP – PRedicting Out-of-OFfice Blood Pressure, SBP – systolic blood pressure.

Notes: The figure shows the proportion of participants that would be referred for out-of-office BP measurement in **Panel A** and the misclassification rate among patients not referred for out-of-office BP measurement in **Panel B** across predicted out-of-office BP thresholds. The optimal predicted out-of-office BP threshold was the one that referred the fewest patients for out-of-office BP measurement with a misclassification rate among those not referred <20%. Misclassification rate is defined as: (false positives + false negatives)/total not referred for out-of-office BP measurement.

**Figure S9. Out-of-office BP measurement referral and misclassification rate with PROOF-BP-US.**

| **A) Proportion recommended for out-of-office BP measurement** | **B) Misclassification rate among participants not recommended for out-of-office BP measurement** |
| --- | --- |
| **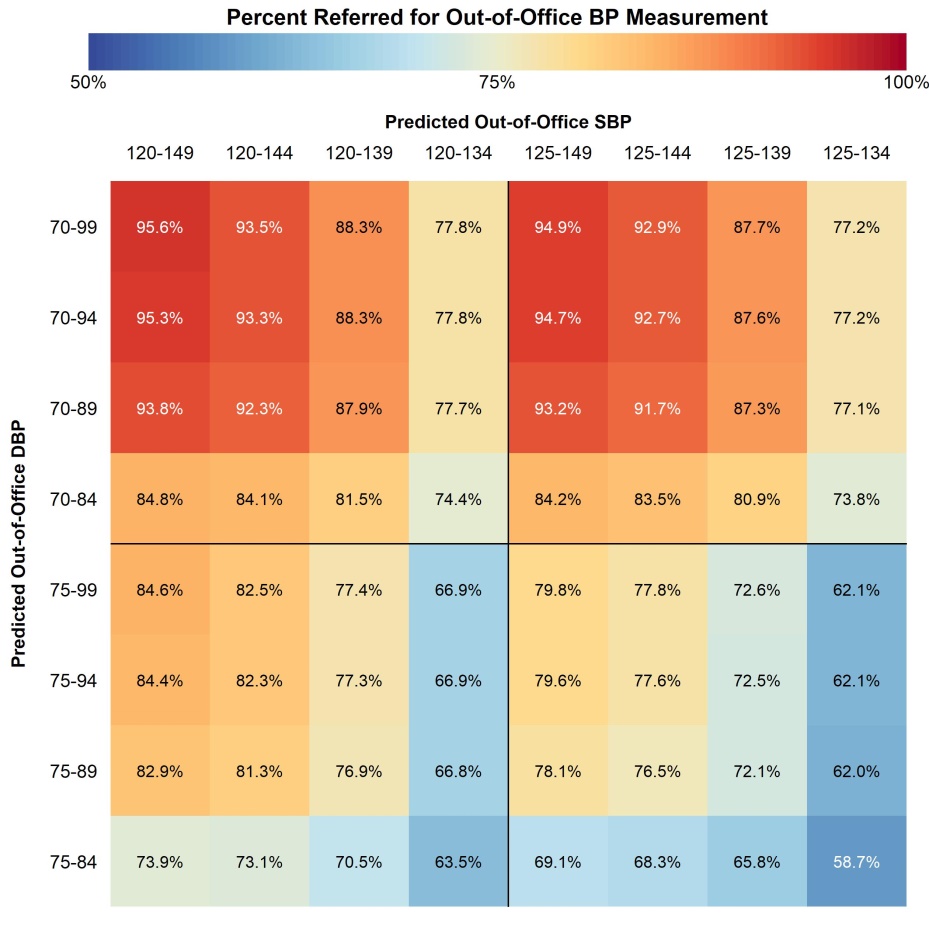** | **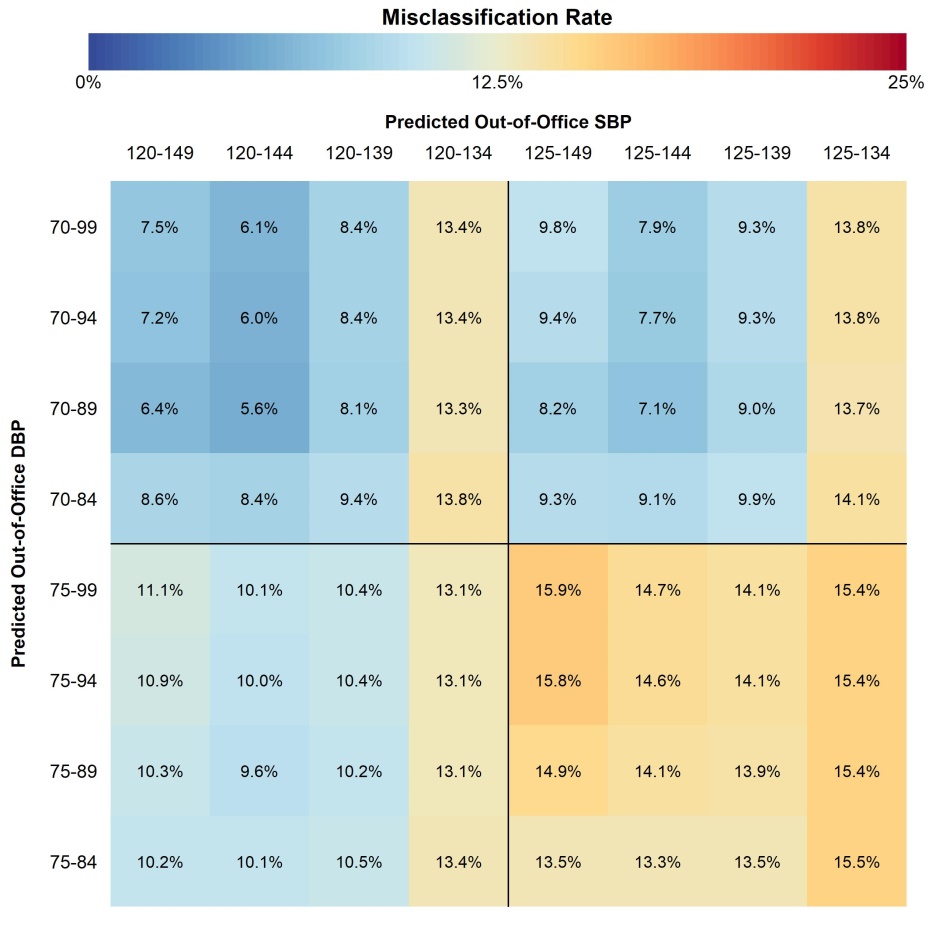** |

BP – blood pressure, DBP – diastolic blood pressure, PROOF-BP – PRedicting Out-of-OFfice Blood Pressure, SBP – systolic blood pressure.

Notes: The figure shows the proportion of participants that would be referred for out-of-office BP measurement in **Panel A** and the misclassification rate among patients not referred for out-of-office BP measurement in **Panel B** across predicted out-of-office BP thresholds. The optimal predicted out-of-office BP threshold was the one that referred the fewest patients for out-of-office BP measurement with a misclassification rate among those not referred <20%. Misclassification rate is defined as: (false positives + false negatives)/total not referred for out-of-office BP measurement.

**SUPPLEMENTAL TABLES**

**Table S1. Covariates included in PROOF-BP and candidate covariates considered for PROOF-BP-US.**

| **Variables** | **PROOF-BP** | | **PROOF-BP-US Candidates** |
| --- | --- | --- | --- |
|  | **SBP difference** | **DBP difference** |  |
| Age | x | x | x |
| Male | x | x | x |
| Race/Ethnicity (non-Hispanic Black vs. other) |  |  | x (sensitivity analysis) |
| Education (high school graduate vs. not) |  |  | x |
| Office BP 1^st^ reading | x | x | x |
| Office BP change from last to 1^st^ reading^a^ | x | x | x |
| Pulse pressure (1^st^ SBP reading – 1^st^ DBP reading) | x | x | x |
| Diagnosed hypertension | x | x | x |
| Years since hypertension diagnosis | x |  | x |
| Antihypertensive medication use | x | x | x |
| Body mass index | x | x | x |
| History of cardiovascular disease events |  | x | x |
| Smoking status (never, former, current) |  |  | x |
| Alcohol consumption (any in last year vs. none) |  |  | x |
| Chronic Kidney Disease |  |  | x |
| Diabetes |  |  | x |
| *Interactions* |  |  | Tested all pairwise interactions with age, sex, and hypertension diagnosis |
| Age x office BP 1^st^ reading | x |  |  |
| Age x pulse pressure | x |  |  |
| Age x office BP change from last to 1^st^ reading^a^ |  | x |  |
| Age x body mass index |  | x |  |
| Age x history of cardiovascular disease events |  | x |  |
| Age x antihypertensive medication use |  | x |  |
| Male x body mass index | x |  |  |
| Male x diagnosed hypertension | x | x |  |
| Male x years since hypertension diagnosis | x |  |  |
| Male x antihypertensive medication use | x | x |  |
| Squared continuous variables |  |  | x |

BP – blood pressure, DBP – diastolic blood pressure, PROOF-BP – PRedicting Out-of-OFfice Blood Pressure, SBP – systolic blood pressure.

^a^Last reading was the third blood pressure (BP) reading in all cohorts except in the Jackson Heart Study, which only had two BP readings per participant.

**Table S2. 2017 ACC/AHA out-of-office BP measurement recommendations.**

| **Antihypertensive Medication Use** | **Recommend Out-of-Office BP Measurement** | | |
| --- | --- | --- | --- |
|  | **Office BP** | **Additional Criteria** | **Screen For** |
| No | SBP 130-159 mmHg and DBP <100 mmHg  *OR*  SBP <160 mmHg and DBP 80-99 mmHg | None | White coat hypertension |
| No | SBP 120-129 mmHg and DBP <80 mmHg  *OR*  SBP <130 mmHg and DBP 75-79 mmHg | None | Masked hypertension |
| Yes | SBP 130-139 mmHg and DBP <90 mmHg  *OR*  SBP <140 mmHg and DBP 80-89 mmHg | Taking multiple (≥3) antihypertensives | White coat effect |
| Yes | SBP <130 mmHg and DBP <80 mmHg | High CVD risk^a^ | Masked uncontrolled hypertension |

ACC – American College of Cardiology, AHA – American Heart Association, BP – blood pressure, CVD – cardiovascular disease, DBP – diastolic blood pressure, SBP – systolic blood pressure.

^a^Defined as a history of cardiovascular disease events, chronic kidney disease, diabetes mellitus, age ≥75 years, or a 10-year atherosclerotic cardiovascular disease risk score ≥10%.

**Table S3. PROOF-BP-US beta coefficients from linear regression models estimating the difference between out-of-office BP and first office BP reading.**

|  | **SBP** | | **DBP** | |
| --- | --- | --- | --- | --- |
| **Variable** | **Beta Coefficient** | **95%CI** | **Beta Coefficient** | **95%CI** |
| Intercept | 37.674 | 17.487, 57.407 | 33.155 | 29.644, 37.553 |
| Age | 0.078 | 0.040, 0.118 | 0.607 | 0.465, 0.720 |
| Male (REF: Female) | 3.808 | 2.930, 4.677 | -0.286 | -2.68, 2.172 |
| Office SBP 1st reading | -0.138 | -0.453, 0.181 | - | - |
| Office SBP change (last minus 1st reading)^a^ | 0.309 | 0.251, 0.369 | - | - |
| Office DBP 1st reading | - | - | -0.564 | -0.593, -0.534 |
| Office DBP change (last minus 1st reading)^a^ | - | - | 0.242 | 0.182, 0.306 |
| Pulse pressure (SBP - DBP) | - | - | 0.074 | 0.033, 0.108 |
| Self-reported hypertension | 4.982 | 3.152, 6.447 | 3.802 | 1.012, 6.246 |
| Antihypertensive medication use | 0.471 | -1.890, 2.677 | - | - |
| Smoking status (REF: never smoker) |  |  |  |  |
| Former | 1.291 | 0.201, 2.399 | 1.000 | 0.332, 1.805 |
| Current | 5.371 | 3.738, 7.440 | 1.954 | 0.951, 3.183 |
| Body mass index | - | - | -0.242 | -0.289, -0.200 |
| Diabetes | 2.959 | 1.783, 4.059 | - | - |
| Age squared | - | - | -0.006 | -0.007, -0.005 |
| Office SBP 1st reading squared | -0.002 | -0.003, 0.000 | - | - |
| Male x smoking status |  |  |  |  |
| Former | -2.258 | -4.075, -0.716 | - | - |
| Current | -2.275 | -4.679, 0.469 | - | - |
| Male x body mass index | - | - | 0.118 | 0.031, 0.201 |
| Self-reported hypertension x pulse pressure | - | - | -0.047 | -0.093, 0.004 |
| Self-reported hypertension x antihypertensive medication use | -3.875 | -6.813, -1.034 | - | - |
| Self-reported hypertension x smoking status |  |  |  |  |
| Former | - | - | -0.990 | -2.341, 0.268 |
| Current | - | - | 2.302 | 0.286, 4.144 |
| Self-reported hypertension x alcohol use | - | - | 1.322 | 0.210, 2.540 |

BP – blood pressure, DBP – diastolic blood pressure, SBP – systolic blood pressure, 95%CI – 95% confidence interval.

^a^Last reading was the third blood pressure (BP) reading in all cohorts except in the Jackson Heart Study, which only had two BP readings per participant.

Notes: The table shows the final multivariable linear regression models to predict the difference between the first office BP reading and the observed awake ABPM. The value predicted by the regression model is then added to the first office BP reading to predict the out-of-office BP. The accelerated bias-corrected 95% confidence intervals for each model were calculated using 500 bootstrap replications.

**Table S4. Sensitivity analysis: PROOF-BP-US beta coefficients from linear regression models estimating the difference between out-of-office BP and first office BP reading when including race as a potential predictor.**

|  | **SBP** | | **DBP** | |
| --- | --- | --- | --- | --- |
| **Variable** | **Beta Coefficient** | **95%CI** | **Beta Coefficient** | **95%CI** |
| Intercept | 37.674 | 17.487, 57.407 | 35.122 | 31.5, 39.458 |
| Age | 0.078 | 0.04, 0.118 | 0.490 | 0.309, 0.641 |
| Male (REF: Female) | 3.808 | 2.93, 4.677 | 2.224 | 1.527, 2.795 |
| Race Black (REF: Non-Black) |  |  | 0.655 | -0.061, 1.432 |
| Office SBP 1st reading | -0.138 | -0.453, 0.181 | - | - |
| Office SBP change (last minus 1st reading)a | 0.309 | 0.251, 0.369 | - | - |
| Office DBP 1st reading | - | - | -0.565 | -0.593, -0.533 |
| Office DBP change (last minus 1st reading)a | - | - | 0.244 | 0.188, 0.308 |
| Pulse pressure (SBP - DBP) | - | - | 0.047 | 0.018, 0.075 |
| Self-reported hypertension | 4.982 | 3.152, 6.447 | 2.460 | 1.389, 3.476 |
| Antihypertensive medication use | 0.471 | -1.89, 2.677 | 6.562 | 1.475, 11.301 |
| Smoking status (REF: never smoker) |  |  |  |  |
| Former | 1.291 | 0.201, 2.399 | 0.889 | 0.171, 1.552 |
| Current | 5.371 | 3.738, 7.44 | 1.576 | 0.566, 2.748 |
| Alcohol use | 1.490 | 0.725, 2.347 | 0.860 | 0.325, 1.461 |
| Body mass index | - | - | -0.210 | -0.253, -0.173 |
| Diabetes | 2.959 | 1.783, 4.059 | - | - |
| Age squared | - | - | -0.005 | -0.006, -0.003 |
| Office SBP 1st reading squared | -0.002 | -0.003, 0 | - | - |
| Male x smoking status |  |  |  |  |
| Former | -2.258 | -4.075, -0.716 | - | - |
| Current | -2.275 | -4.679, 0.469 | - | - |
| Self-reported hypertension x antihypertensive medication use | -3.875 | -6.813, -1.034 | - | - |
| Self-reported hypertension x smoking status |  |  |  |  |
| Former | - | - | -0.874 | -2.228, 0.438 |
| Current | - | - | 2.332 | 0.336, 3.958 |
| Age x hypertension medication use | - | - | -0.129 | -0.203, -0.046 |
| Male x race Black | - | - | 1.931 | 0.964, 2.953 |

BP – blood pressure, DBP – diastolic blood pressure, SBP – systolic blood pressure, 95%CI – 95% confidence interval.

^a^Last reading was the third blood pressure (BP) reading in all cohorts except in the Jackson Heart Study, which only had two BP readings per participant.

Notes: The table shows the results of the multivariable linear regression models predicting the difference between the first office BP reading and the observed awake ABPM. The value predicted by the regression model is then added to the first office BP reading to predict the out-of-office BP. The accelerated bias corrected 95% confidence intervals for each model were calculated using 500 bootstrap replications.

**REFERENCES**

1. Friedman GD, Cutter GR, Donahue RP, Hughes GH, Hulley SB, Jacobs DR, Jr., Liu K, Savage PJ. CARDIA: study design, recruitment, and some characteristics of the examined subjects. *J Clin Epidemiol.* 1988;41(11):1105-1116. PMID: 3204420.

2. Poudel B, Booth JN, 3rd, Sakhuja S, Moran AE, Schwartz JE, Lloyd-Jones DM, Lewis CE, Shikany JM, Shimbo D, Muntner P. Prevalence of ambulatory blood pressure phenotypes using the 2017 American College of Cardiology/American Heart Association blood pressure guideline thresholds: data from the Coronary Artery Risk Development in Young Adults study. *J Hypertens.* Jul 2019;37(7):1401-1410. PMID: 31145711.

3. Booth JN, Anstey DE, Bello NA, Jaeger BC, Pugliese DN, Thomas SJ, Deng L, Shikany JM, Lloyd-Jones D, Schwartz JE, Lewis CE, Shimbo D, Muntner P. Race and sex differences in asleep blood pressure: The Coronary Artery Risk Development in Young Adults (CARDIA) study. *J Clin Hypertens (Greenwich).* Feb 2019;21(2):184-192. PMID: 30719843. “PMC6375074:” PMC6375074.

4. Sempos CT, Bild DE, Manolio TA. Overview of the Jackson Heart Study: a study of cardiovascular diseases in African American men and women. *Am J Med Sci.* Mar 1999;317(3):142-146. PMID: 10100686.

5. Schwartz JE, Burg MM, Shimbo D, Broderick JE, Stone AA, Ishikawa J, Sloan R, Yurgel T, Grossman S, Pickering TG. Clinic Blood Pressure Underestimates Ambulatory Blood Pressure in an Untreated Employer-Based US Population: Results From the Masked Hypertension Study. *Circulation.* Dec 6 2016;134(23):1794-1807. PMID: 27920072. “PMC5151173:” PMC5151173.

6. Schwartz JE, Muntner P, Kronish IM, Burg MM, Pickering TG, Bigger JT, Shimbo D. Reliability of office, home, and ambulatory blood pressure measurements and correlation with left ventricular mass. *J Am Coll Cardiol.* 2020;76(25):2911-2922. PMID:

7. Johnson CL, Dohrmann SM, Burt VL, Mohadjer LK. National health and nutrition examination survey: sample design, 2011-2014. *Vital Health Stat 2.* Mar 2014(162):1-33. PMID: 25569458.

8. Shimbo D, Newman JD, Schwartz JE. Masked hypertension and prehypertension: diagnostic overlap and interrelationships with left ventricular mass: the Masked Hypertension Study. *Am J Hypertens.* Jun 2012;25(6):664-671. PMID: 22378035. “PMC3668422:” PMC3668422.

9. Sheppard JP, Stevens R, Gill P, Martin U, Godwin M, Hanley J, Heneghan C, Hobbs FD, Mant J, McKinstry B, Myers M, Nunan D, Ward A, Williams B, McManus RJ. Predicting Out-of-Office Blood Pressure in the Clinic (PROOF-BP): Derivation and Validation of a Tool to Improve the Accuracy of Blood Pressure Measurement in Clinical Practice. *Hypertension.* May 2016;67(5):941-950. PMID: 27001299. “PMC4905620:” PMC4905620.
